# A systematic review and meta‐analysis of factors associated with adolescent substance use in Africa, 2000 to 2020

**DOI:** 10.1111/add.70023

**Published:** 2025-02-27

**Authors:** Sandra Jumbe, Tony Mwenda Kamninga, Ukwuori‐Gisela Kalu, Joel Nyali, Lara Saleh, Chris Newby, Joel Msafiri Francis

**Affiliations:** ^1^ School of Social and Health Sciences Millennium University Blantyre Malawi; ^2^ Centre for Evaluation and Methods, Wolfson Institute of Population Health Queen Mary University of London London UK; ^3^ The Medical School University of Nottingham Nottingham UK; ^4^ Department of Family Medicine and Primary Care, Faculty of Health Sciences University of Witwatersrand Johannesburg South Africa

**Keywords:** adolescent, Africa, factors, meta‐analysis, substance use, systematic review

## Abstract

**Background and aims:**

Adolescent substance use is a growing public health concern in Africa, yet little is known about the contextual factors of substance use among young African populations. This systematic review identified factors associated with substance use among adolescents (aged 10–19 years) in Africa.

**Methods:**

This review was conducted in line with PRISMA guidelines. We searched six databases (from January 2000 to December 2020): PubMed, Cochrane Library, African Journals Online (AJOL), Google Scholar, ScienceDirect and World Health Organization (WHO) African Index Medicus. We included population‐based observational studies reporting on factors associated with adolescent substance use across Africa. Study screening was conducted by at least four independent reviewers who resolved discrepancies through discussion and consensus. All included studies were analysed in a narrative synthesis. Studies providing sufficient statistics, i.e. three or more reporting the same outcome and exposure/predictor, were included in meta‐analyses.

**Results:**

Sixty‐three peer reviewed studies that were full text accessible were included. The majority were cross‐sectional surveys. Factors associated with adolescent substance use identified were linked to individual, family, socioenvironmental and non‐familial social networks determinants. Results from both the narrative synthesis and meta‐analysis revealed that being male and an older adolescent were significantly associated with adolescent substance use. Combined odds ratio (OR) of males who currently smoke compared with females was 1.81 [95% confidence interval (CI) = 1.37–2.39; 6 studies, 13 443 participants, I^2^ = 59.67%]. Additional meta‐analysis outcomes found that having a friend who smokes was associated with tobacco smoking. Combined OR of ‘ever‐smokers’ with a friend who smoked was 4.83 (CI = 2.56–9.10; 3 studies, 18 858 participants, I^2^ = 79.21%). Having a family member who smokes was associated with smoking initiation (OR = 2.99; CI = 2.67–3.35; 3 studies, 18 858 participants, I^2^ = 0%) and current smoking (OR = 2.33; CI = 2.23–2.45; 4 studies, 13 282 participants, I^2^ = 0%).

**Conclusion:**

Multiple factors that operate on individual, family and societal levels influence adolescent substance use in Africa. Key factors of adolescent substance use in Africa appear to include being male, being an older adolescent and being exposed to peer substance use.

## INTRODUCTION

Adolescents, defined as people age 10 to 19 years [1], often find that their health needs are neglected despite representing a fifth of the global population because they are perceived to be healthy [2, 3]. Substance use that includes alcohol, tobacco and illicit drug use is common during adolescence [4]. Young people who misuse alcohol and drugs experience more health issues such as weight loss, headaches, sleep disturbance and depression than their counterparts [3, 5], resulting in negative effects on their learning and development [6]. In this article, substance misuse refers to the use of illegal drugs and the inappropriate use of legal substances like alcohol and tobacco. Since 2000, the number of years lost to disability because of mental and substance use disorders in Africa has increased by 52% [7]. A recent systematic review found an overall prevalence of ‘any substance use’ among adolescents in sub‐Saharan African is 41.6%, with alcohol and tobacco being the highest prevailing substances (i.e. 40.8% and 45.6%, respectively) across the continent compared to any other substance use [8]. Other studies have found substance use among young people to be associated with psychosocial issues like violence, vandalism, theft, risky sexual behaviour, self‐harm and impaired relationships with family and friends [9, 10, 11].

To our knowledge, there is no systematic review that provides an accurate understanding of the determinants of adolescent substance use in Africa. By determinants, we refer to a range of factors that influence substance use among individuals or populations. Several studies conducted in Africa found that having family or friends who use substances is significantly associated with substance use [10, 12] along with childhood trauma and adverse experience like physical, emotional and sexual abuse [13]. Other factors includes demographic and socio‐economic factors such as being male [14], initiation at younger age, lower education grades, having divorced parents, unemployed or fully employed mothers [15]. However, because these studies are typically country specific, a more extensive holistic analysis is necessary to pool together evidence and situate the factors associated with substance use across a broader spectrum cultures and settings within the African continent.

Adolescent substance use is a growing major public health concern in Africa [8]. Knowledge on the true extent and determinants of this phenomenon needs to be understood across the various cultural settings of the continent, while considering socioenvironmental factors, competing health priorities and limited treatment options [3, 7]. Despite the increasing prevalence and increased risks of severe health, economic and social problems related to substance use, consolidated evidence about the social contexts of adolescent substance use in Africa is lacking [9]. It is evident that harmful adolescent substance use is because of complex multilevel interactions of risk and protective factors, influenced by an individual's development, experience, and health, together with one's relationship with family, community environment and broader societal‐level factors like culture, policy and socio‐economic status (SES) [16]. Pooling together knowledge and research evidence on factors associated with adolescent substance use in Africa will vitally inform development of effective prevention and treatment strategies, enhancing the achievement of Sustainable Development Goals (SDGs) 3.5 and 3.4 1 for the region [8, 17]. Effectively tackling substance use among young people in Africa where negative outcomes are more common would bring great benefit to human health.

This systematic review and meta‐analysis aimed to identify and evaluate qualitative and quantitative epidemiological studies describing factors associated with substance use among adolescents age 10 to 19 years old in Africa [18]. The main outcome of this review was to characterise key factors that significantly influence any adolescent substance use in Africa, including study‐level variables like gender and (mental) health status and regional comparisons where possible.

## METHODS

**FIGURE 1:**
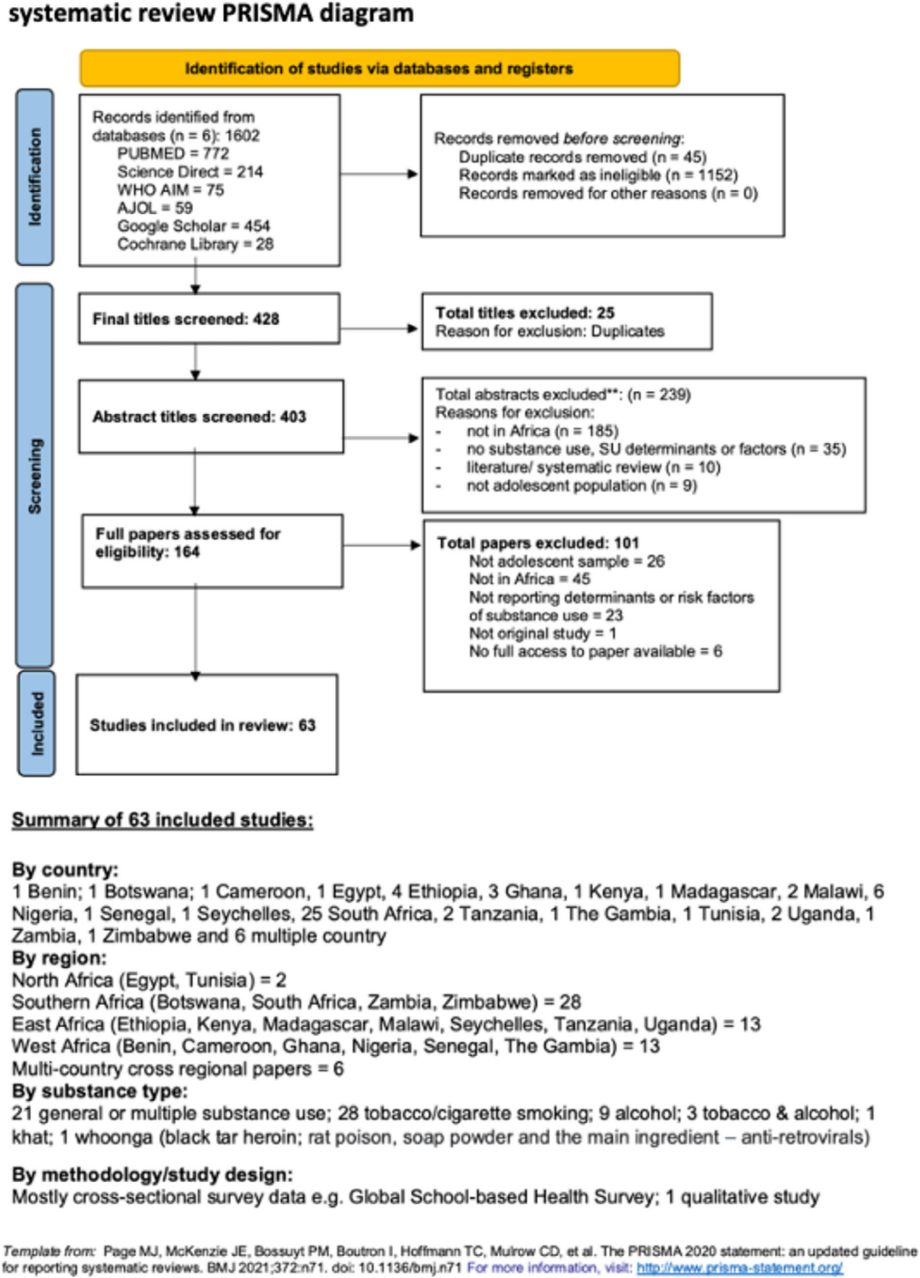
Factors associated with adolescent substance use in Africa (FASA) systematic review Preferred Reporting Items for Systematic Reviews and Meta‐Analyses (PRISMA) diagram.

Before commencing this systematic review, a detailed protocol of review work to be done was registered (PROSPERO CRD42020190158) and subsequently published [18]. In accordance with the Preferred Reporting Items for Systematic Reviews and Meta‐Analyses (PRISMA) guidelines, we implemented a four‐step process to select studies for inclusion in the review [19]. In brief, relevant studies were identified through extensive literature searches on the following databases: PUBMED, Science Direct, World Health Organization (WHO) AIM, African Journals Online, Google Scholar and the Cochrane Library (see Figure 1, PRISMA flow diagram), published from January 2000 to December 2020. Second, searches contained keyword combinations of terms related to factors, adolescent, substance use and Africa. These were agreed on by the team based on a quick scoping of initial ‘dummy’ searches and previous reviews. A draft search strategy for PubMed/MEDLINE is provided in the published protocol [18]. We also conducted a search on Google Scholar and hand‐searching of reference lists of included studies, relevant reviews and other relevant documents/grey literature to ensure no key empirical literature was missed.

### Inclusion and exclusion criteria

Studies were included if they were empirical studies involving participant samples of adolescents age 10 to 19 years old, according to the WHO definition [1, 20], conducted in Africa, reporting factors associated with substance use and published in English or French. There were few cases where the age groups intersected with age brackets outside 10 to 19, for instance 15 to 24 years old [21, 22]. We excluded such articles unless the results in the article were only showing for those that had our age of interest. Both quantitative (e.g. cohort, cross‐sectional or health surveys) and qualitative studies were included. We excluded studies that used non‐human samples, reviews and methodological studies.

### Quality assessment

Included articles were divided among four reviewers (co‐authors J.N., S.J., T.M. and U.K.) to independently evaluate using the Effective Public Health Practice Project (EPHPP) tool for assessing the quality for quantitative studies [23]. This tool was chosen for its ability to extensively assess methodological quality across different quantitative research designs. Presence of selection bias and confounders, study design, blinding, data collection methods, withdrawals and drop‐outs, and the appropriateness of the study's analysis to the research question were assessed [23]. The Critical Appraisal Skills Programme (CASP) was used to assess the rigorousness, credibility, and relevance of the one included qualitative study [24].

Two reviewers (co‐authors S.J. and U.K.) ultimately assessed each study independently before its inclusion in the final review using the relevant checklist, with studies being graded to assess quality of the study design and reporting. Discrepancies in reviewers' evaluations were discussed until consensus was reached. No disagreements arose between reviewers' ratings that required resolution by a third reviewer.

### Data extraction and synthesis

Four authors (J.N., S.J., T.M. and U.K.) extracted data and methods from the included studies using a custom‐made data extraction form, later embedded onto a REDCap database. Data abstraction included study samples (e.g. sample type, size and groupings), study setting, research focus, study design, sample size, participants' age mean/range, substance type (e.g. alcohol, tobacco), determinants (reported factors associated with substance use) and a summary of results (direction, strength and significance of a factor associated with adolescent substance use). S.J. reviewed and cross‐checked data from all articles extracted by the other reviewers to gauge consistency and minimise issues of missing data.

Characteristics of the included studies are presented in Table 1. We conducted a narrative synthesis of results from the included studies. This involved using a textual approach to analyse relationships within and between the included studies to provide an overall assessment of the evidence focusing on reported associations between each factor and adolescent substance use. Our systematic review question included any type of adolescent substance use across Africa. Therefore, a narrative synthesis was an ideal approach to facilitate textual analysis of rich heterogeneous data of studies that could not be included in the meta‐analysis. This methodological approach provided flexibility to systematically synthesise key findings from studies with diverse aims, designs and methodologies [25]. We took on an inductive approach to our narrative analysis where patterns and themes were derived from the data without preconceived theories or categories. Where possible, regional comparisons of key findings across the continent are included.

**TABLE 1:** Study characteristics based on study type, sampling and data collection methods.

Factor	Frequency	Percentage
Type of studies		
Cross‐sectional	49	78
Cohort	8	13
Randomized controlled trial	2	3
Case control study	1	2
Qualitative	1	1
Unspecified	2	3
Cumulative	63	100
Sampling methods		
Unspecified	9	14
Random sampling	33	52
Purposive sampling	6	10
Stratified sampling	13	21
Snowball sampling	2	3
Cumulative	63	100
Data collection methods		
Questionnaire	53	84
In‐depth interview assessment	2	3
Focus group discussion	1	2
Medical records	4	6
Other	3	5
Cumulative	63	100

### Selection of studies for meta‐analyses

All included articles for this systematic review were reviewed for possible meta‐analysis. Studies were deemed appropriate for meta‐analysis if three or more articles were found with the same outcome and exposure/predictor, as just two studies can be better summarised in the narrative synthesis. Articles were screened by all authors to record outcome and predictor/exposure, with final oversight by co‐author C.N. Specifically, this was a two‐stage process. First outcomes from included articles for multiple substances (e.g. ever smoked cannabis? Yes/no), alcohol (e.g. ever drank alcohol? Yes/no) and smoking (e.g. current smoker? Yes/no) were recorded. Second, each outcome recorded in more than three articles was taken forward to explore the number of exposures/predictors in those subsets of articles to determine if the three article criteria of both same exposure/predictor and outcome were met for meta‐analysis.

Estimates were calculated with 95% confidence intervals (CI). Pooled effect sizes (e.g. proportions, ORs) of identified factors associated with substance use with 95% CI from included quantitative studies were calculated using a random‐effects model, based on expected heterogeneity of the included diverse populations. Heterogeneity between the studies was assessed using the I^2^ statistic, which describes percentage of variation across studies that is because of heterogeneity rather than chance. I^2^ value greater than 50% was considered indicative of substantial heterogeneity. All analysis was carried in R language with the package.

## RESULTS

### Summary of search results

The search from six electronic databases yielded a total of 1602 records. A manual search of relevant reviews and other relevant documents' reference lists yielded no additional studies. After titles' screening and removing duplicates, 403 records were available for abstract screening. Of these, 164 studies went forward for full‐text review. After screening of the 164 full text articles was done, 101 studies were excluded because they were not based in Africa (*n* = 45), they did not have an adolescent sample (*n* = 26) or they did not report factors of substance use (*n* = 23). One article was not an original study. We failed to get full access to six articles despite multiple emails to authors and requests to institutional libraries to source them. A total of 63 studies were found to meet the inclusion criteria and were included in the review (see Figure 1).

### Characteristics of included studies

Characteristics of studies are summarised in Table 1 and further details including quality assessment ratings are in Table S1. Twenty‐four studies were published between 2000 and 2009, and 39 (62%) were published between 2010 and 2020. Most of the included studies (*n* = 28) were conducted in Southern Africa, whereas only two studies were from North Africa [26, 27]. The rest were split evenly between East (*n* = 13) and West Africa (*n* = 13). Fifty‐six of the included studies focused on a single country, whereas seven studies combined samples across two or more countries. One study looking at second hand smoke exposure and susceptibility to cigarette smoking initiation used the Global Youth Tobacco Survey (GYTS) data from 29 African countries [28]. Twenty‐eight studies targeted tobacco‐use (44%), nine studies targeted alcohol‐use (14%) and one study investigated Whoonga use [29] and another khat use [30]. The rest of the studies looked at mixed substance use (i.e. two or more). Thirty studies disclosed sources of research funding (48%), five received no funding for their research and 28 studies did not have any statements on research funding (44%).

Table 1 below details study design characteristics of the 63 included studies. The majority of studies (*n* = 49) used a cross‐sectional study design typically involving questionnaire‐based survey data. Common questionnaires used were the Global School‐Based Student Health Survey (GSHS) and GYTS. There was only one qualitative study [31].

### Sample characteristics

Age was reported differently in the studies; therefore, a mean age cannot be reported across all studies. All included studies' samples had both males and females participating. Thirty eight of 63 (60%) of the population were school going students. There were a few studies with more unique populations. For example, Cumber and Tsoka‐Gwegweni [32] and Embleton *et al*. [33] studied street children who were homeless and living on the streets in Cameroon and Kenya, respectively. Meanwhile Birungi *et al*. [34] looked at substance use risk factors among adolescents with HIV in Kampala, Uganda. There were 10 studies, all from South Africa, that reported on ethnicity or race of their participants, typically using these three categories i.e. Blacks, Coloured and White [31, 35, 36, 37, 38, 39, 40, 41, 42, 43].

### Quality of included studies

Table S1 shows the methodological quality of the studies used to identify the determinants and risk factors of adolescent substance use. Methodological quality for the 62 quantitative studies was assessed using the EPHPP tool [23]. A total of 21% of the studies were considered strong (*n* = 13), and 23% considered moderate (*n* = 14), indicating weak methodological quality for most included studies (*n* = 35). According to the CASP, the only qualitative study was rated as moderate [24]. All studies focused on adolescents age 10 to 19 and referred to at least one or more substances (e.g. tobacco, alcohol and cannabis). All studies reported that ethical approval was obtained before any participant recruitment.

### Major themes of factors associated with adolescent substance use—Narrative synthesis

**FIGURE 2:**
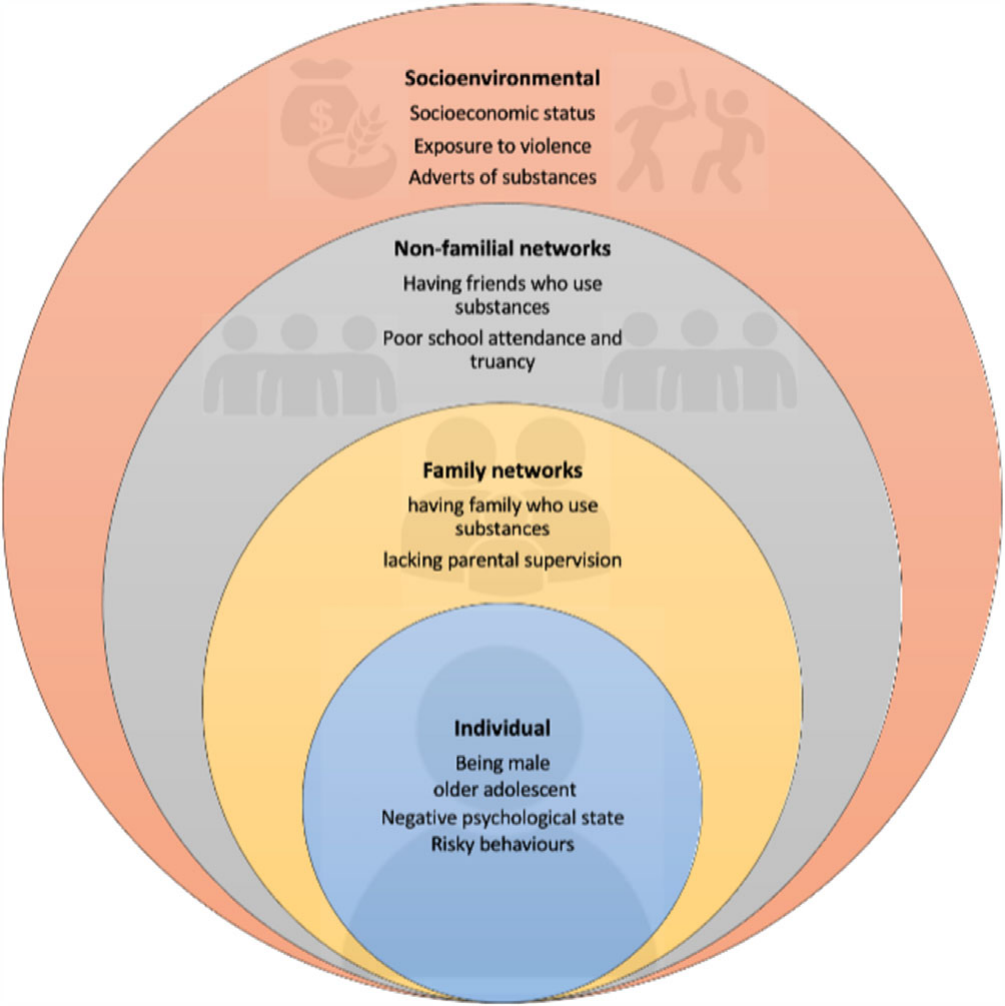
Thematic map of factors associated with adolescent substance use.

All the key dominant factors associated with adolescent substance use identified from included studies are listed in Supporting information. These wide‐ranging factors reported in the included studies are linked to one of four categories of the socio‐ecological model (SEM), namely individual, family, socio‐economic or environmental and non‐familial social networks. As part of our narrative synthesis, we identified dominant factors presented in Figure 2 using inductive narrative analysis of data from the included studies and categorised them into themes that were subsequently linked to one of the aforementioned four categories of SEM. These are discussed below in more detail. We have categorised a ‘dominant factor’ as one that was reported in three or more studies per substance type, like the meta‐analysis criteria. Where relevant, themes are backed by meta‐analysis outcomes.

### Individual level factors

Individual level factors are a person's fixed biological and genetic characteristics (e.g. age, sex and family history of disease) that we cannot control and personal behaviours (exercise, diet and unprotected sex) that can increase the risk of the reference disease or health condition [44]. Three themes were identified for the individual level factors associated with adolescent substance use, namely: (1) being male and an older adolescent; (2) negative psychological state; and (3) high risk behaviours.

#### Theme 1: Being male and an older adolescent

Gender and age were reported as important contributors to adolescent substance use. Specifically, all 38 of 63 articles found that being male was significantly associated with adolescent substance use [45, 46, 47, 48] with males more likely to use substances than females.

Twenty‐seven of 63 included articles reported age as a significant factor associated with substance use among adolescents. Specifically, all 27 articles reported that older adolescents were more likely to use substances [45, 48, 49, 50]. These articles included varied substances (alcohol, tobacco, *etc*) and were from countries across all regions in Africa except for the North (i.e. 6 in East Africa, 5 in West Africa and 16 in Southern Africa).

#### Theme 2: Negative psychological state

Nineteen of the 63 included articles reported either being depressed, also known as low mood, sad, hopeless and/or having anxiety as factors significantly associated with adolescent substance use. Nine of these articles reported both depression and anxiety. Another nine articles reported depression exclusively, whereas only one article by Oppong‐Asante and Kugbey [51] that explored alcohol use by school‐going adolescents in Ghana reported anxiety alone. These articles included studies looking at varied substances (e.g. cannabis, alcohol, tobacco and multiple substances) and were from countries across regions on the continent. Six articles also reported suicide ideation (i.e. thoughts of suicide and wanting to take one's own life), planning and/or suicide attempt to be associated with substance use among adolescents [51, 52, 53, 54, 55]. In terms of substance type, one of these articles was on cannabis, two were on alcohol, another one was on tobacco and the last two articles involved multiple substances. These studies were conducted in countries across east, west and southern regions. Three of the six articles that found a link between suicide and adolescent substance use also found loneliness to be associated with increases substance use although suicidal ideation had a stronger association [54]. For instance, in Oppong Asante and Kugbey [51], loneliness, suicidal ideation and suicidal attempts independently increased the odds of alcohol use behaviours (current alcohol use, lifetime drunkenness and alcohol problems) among adolescents. Oppong Asante [56] also found loneliness and suicidal behaviour (i.e. ideation, plan and attempt) were related to cannabis use.

#### Theme 3: High risk behaviours

High risk behaviours were also linked to adolescent substance use within a subset of the included studies. Typical examples of risky behaviours (i.e. acts that increase risk of disease or harm) were binge drinking or unprotected sex. A total of eight articles from the included studies reported hazardous drug or alcohol use. Of these eight articles, five found both hazardous drug and alcohol use. Two of eight articles reported hazardous alcohol use exclusively [57, 58] and one other article reported only drug use [14]. Concomitant drug use (two or more drugs used or given at or almost at the same time) was another crucial feature among 15 articles. In Oppong Asante and Kugbey [51] and Siziya [52] 2009 (alcohol use articles), those who smoked cigarettes were more likely to use alcohol among school‐going adolescents. Similarly, Oppong Asante [56] found that school‐going adolescents who smoke cigarettes were more likely to use cannabis and amphetamine.

### Family determinants

Several significant family factors emerged, with two themes identified namely [1] having family who use substances and [2] lacking parental supervision.

#### Theme 1: Having family who use substances

Adolescents' substance use was linked to having a family member who uses a substance (i.e. tobacco smoking or drinking alcohol in 25 of the included articles). Adolescents with parents who used substances were more likely to use substances [48, 59, 60, 61]. The majority of these studies (*n* = 15) reported on tobacco smoking or use, two were on alcohol [46, 51] and eight included multiple substances [32, 33, 57, 62, 63, 64, 65, 66]. In 10 articles, adolescents who lived with someone who uses a substance use was also more likely to use the substance. Seven of these articles were on tobacco use [27, 47, 49, 67, 68, 69, 70], whereas the others were on alcohol [46], khat chewing [30] and psychoactive substance use [63]. The Kabiru [46] study included data from four countries namely Burkina Faso, Ghana, Malawi and Uganda.

#### Theme 2: Lacking parental supervision

Lacking parental supervision was associated with adolescent substance uses among adolescents in 12 articles. This finding was consistent in articles investigating multiple substances (*n* = 6) and also reported in five articles exclusively looking at smoking or tobacco use [31, 38, 53, 60, 71] and one alcohol article [52]. These 12 articles were conducted in countries across all regions of the African continent.

### Socio‐environmental determinants

Three themes were identified that described a link between adolescent substance use with social, economic and environmental risk factors: (1) SES; (2) exposure to violence; and (3) advertisements of substances.

#### Theme 1: SES

Few articles explored SES as a factor associated with adolescent substance use. In Ramsoomar *et al*. [45], SES was significantly associated with adolescent alcohol use in that low SES was related to higher alcohol use. Kapito‐Tembo *et al*. [26] and Harrabi *et al*. [61] found contrasting results with tobacco smoking. In their case, adolescents who received pocket money were more likely to be current smokers than those who did not receive pocket money. The higher the monthly pocket money the higher the likelihood of the adolescent to be a current smoker. Itanyi *et al*. [60] also found that in schools set in rural areas, high SES increased the odds of smoking (OR = 1.42; 95% CI = 1.03–1.96), but this was not the case in urban schools. Students of high SES in schools in urban areas had lower odds of being current smokers.

#### Theme 2: Exposure to violence

Exposure to violence [55, 72, 73], physical abuse [46] and reporting history of abuse or neglect [64] was associated with substance use among adolescents in few articles. In Kabiru *et al*. [46], twice as many respondents reporting physical abuse in childhood (12%) reported that they had been drunk compared to those reporting no physical abuse (6%). In Magidson *et al*. [72], exposure to violence was associated with alcohol use for both male and female adolescents. The high rates of violence associated with substance use in this population may reflect adolescent use as a coping strategy for experiences of violence.

#### Theme 3: Advertisements of substances

Results from several included articles suggested that exposure to advertisements of alcohol or tobacco brands, advertising and products were significantly associated with substance use among adolescents [36, 60, 69, 73, 74, 75]. Exposure to tobacco industry advertisements and promotions was associated with increased tobacco use [75, 76]. In Kaduri *et al*. [77], a large proportion of the smokeless tobacco users first knew about smokeless tobacco via advertisement. In Veeranki *et al*. [48] and Rudatsikira *et al*. [69], exposure to tobacco industry promotions was significantly associated with increased probability of smoking initiation at an early age (before 10 years old). In addition, six articles found that specifically liking any advertisements or promotions of tobacco or alcohol related substances were a significant factor for adolescent substance use [48, 60, 69, 75, 76, 77, 78].

### Non‐familial social networks determinants

Two themes were identified under this determinant, namely (1) having friends who use influences substance use; and (2) poor school attendance and truancy.

#### Theme 1: Having friends who use influences substance use

There were 25 articles that reported having friends who smoke or drink to be associated with substance use among adolescents. For example, Parry and colleagues [50] showed that older adolescents and adolescents whose friends drink were more likely to have been drunk at least once. Similarly, several (*n* = 8) articles (e.g. Muula [49]; Muula and Siziya [47], Odukoya *et al*. [59] and Rudatsikira *et al*. [69]) found that having a friend who was a smoker was strongly associated with increased tobacco use or strongly influenced smoking initiation [70]. In Reda *et al*., [30] chewing khat were eight times higher among students who had friends who chewed khat compared to those who did not (OR = 7.93; 95% CI = 5.40–11.64). Articles that looked at multiple substance use also found that participants who reported having friends who smoke or drink were more likely to substance use [65], particularly regarding initiation of substance use where participants who used were introduced to the substance by close friends [26, 33]. Two articles found that this influence extended to peers [75, 79], with one article finding that students with peers that smoked had a fivefold increase in odds of current smoking [75].

#### Theme 2: Poor school attendance and truancy

Seven articles reported a link between adolescents' attendance to school and substance use. Two articles by Oppong Asante and colleagues found that truancy and substance use (cigarette smoking) were independently associated with both past‐month cannabis use and lifetime amphetamine use [51, 56]. In one study, school truancy (OR = 3.34; 95% CI = 1.88–5.92; *P* < 0.001) and current smoking (OR = 12.48; 95% CI = 6.48–24.02; *P* < 0.001) were associated with past‐month cannabis use [56]. A gender disparity was suggested in some articles suggesting truancy was more associated with substance use among males, but not females [53]. Contrarily, Alwan [54] found truancy (measured by missing school without permission) was strongly associated with cannabis use particularly among girls (OR = 33, 95% CI = 6.9–160.6), although this finding was based on few cases. Three articles also found that adolescents with poor academic performance were more likely to use substances. For instance, in Birhanu *et al*. [66] students who had poor academic performance were 1.67 times more likely to use substances [AOR (95% CI) = 1.67 (1.12–2.47)] than their counter parts who had good academic performance. Likewise, in a study by Atilola and colleagues [57] self‐reported poor academic performance was the most significant factor independently associated with 12‐month alcohol and substance use. In Oshodi *et al*. [14], 3.9% believed that poor academic performance could arise from substance use. However, more worryingly 73% of their respondents did not believe any issues could arise from their substance use habits.

### Meta‐analysis on predictors of adolescent substance use

For the nine articles with alcohol as the main substance type, only three factors, namely age (7 articles), gender (8 articles) and SES (3 articles) featured (Appendix S2). On further inspection of the predictors and outcomes, only two of the nine articles had the same factor and outcome (gender, male/female and alcohol use yes/no). The seven other articles either had different definitions of alcohol outcomes (<3 with the same outcome) or different definitions of factors associated with alcohol use (<2 the same). Therefore, no data was combined for alcohol. This was also the case for multiple substances, with articles usually concentrating on a specific combination of substances that have been explored in the narrative synthesis.

For smoking, the following four outcomes were featured in more than three articles:

1. Ever smoked versus never smoked (12 articles);
2. Current smoker versus not smoking (10 articles);
3. Smoked in the last 30 days (5 articles); and
4. How many cigarettes smoked in last 30 days (3 articles).

For outcomes ‘smoked in the last 30 days’ and ‘how many cigarettes smoked in last 30 days’, although information was featured in the articles on eight predictors across at least three articles, this was often only as summary data or using different definitions of predictors or categories (Appendix S3).

### Ever smoked

For the 12 articles featuring the ‘ever smoked’ outcome, there were initially 13 factors that were mentioned in three or more articles that could be suitable for meta‐analysis. However, closer inspection of these found that only three factors had the same definitions across three or more articles that could be used for meta‐analysis. These were gender, having a parent who smokes and having a friend who smokes (Appendix S3).

**FIGURE 3:**
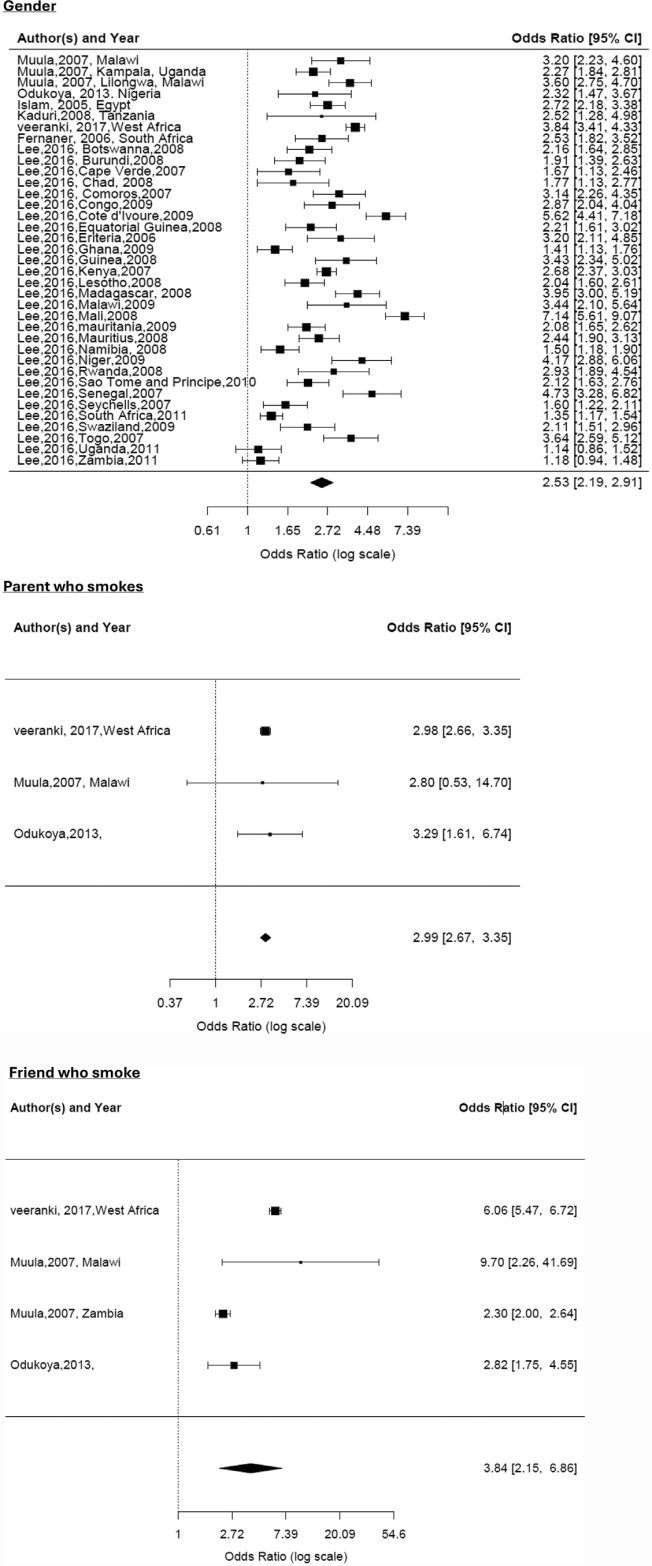
Forest plots for meta‐analysis of articles with ‘ever smoked’ outcome.

For the meta‐analysis on ‘gender’ variable, eight primary research articles [27, 40, 47, 48, 49, 59, 77, 80] were included from our search and an article with data from the GYTS of 29 African countries [28]. Individual country results from the article by Lee and colleagues [28] were included in the meta‐analysis. The combined OR of males ever smoking compared to females ever smoking was 2.53 (CI = 2.19–2.91; 13 443 participants) but there was significant heterogeneity (I^2^ = 90.66%) and there was insufficient data to assess publication bias (Figure 3).

For the ‘parent who smokes’ variable, only three articles were included [48, 59]. The combined OR of people who have a parent who smokes, who ever‐smoked was 2.99 (CI = 2.67–3.35; 18 858 participants) compared to people whose parents did not smoke. Heterogeneity was low (I^2^ = 0%), and there was insufficient data to assess publication bias.

For ‘having a friend who smokes’ variable, only the same three articles were included [48, 59]. The combined OR of people who have a friend who smokes, who ever‐smoked was 4.83 (CI = 2.56–9.10; 18 858 participants) compared to people who did not have a friend who smokes. Heterogeneity was high (I^2^ = 79.21%), but again, insufficient data to assess publication bias.

### Current smoking

For the 10 articles that featured the current smoking outcome, there were 10 factors that were mentioned in three or more articles that could be suitable for meta‐analysis (Appendix S3). Again, closer inspection of these revealed that only two factors had the same definitions across three or more articles that could be used for meta‐analysis. These were gender and having a parent who currently smokes.

**FIGURE 4:**
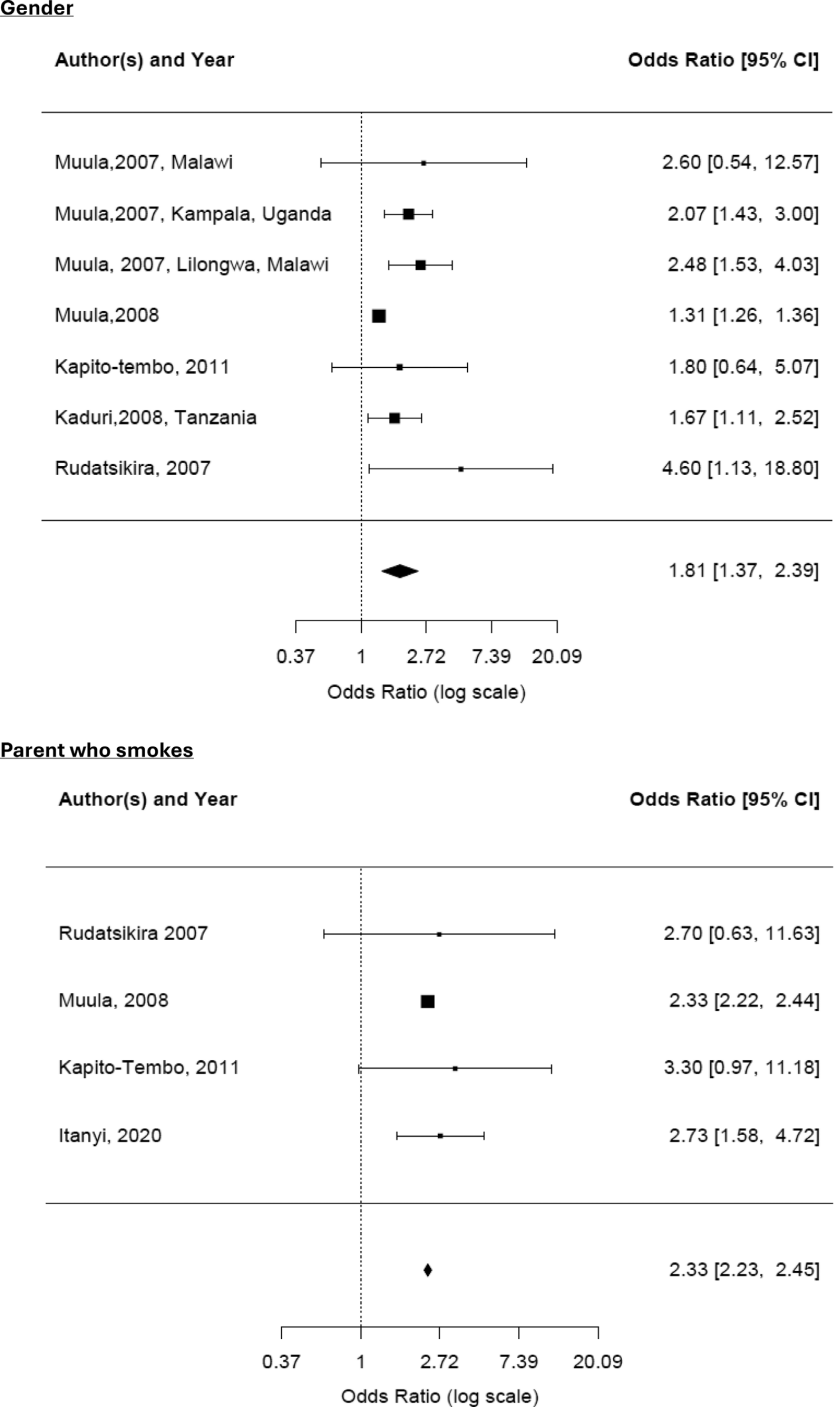
Forest plots for meta‐analysis of articles with ‘current smoker’ outcome.

For the ‘gender’ variable meta‐analysis, seven primary research statistics from six articles were included, namely Muula [47], Muula and Siziya [49], Muula *et al*. [61], Kapito‐Tembo *et al*. [69], Kaduri *et al*. [77] and Rudatsikira [81]. The combined OR of males who currently smoke compared to females was 1.81 (CI = 1.37–2.39; 13 443 participants). Heterogeneity was reasonable (I^2^ = 59.67%). For the ‘parent who smokes’ variable, only four articles were included [60, 61, 69, 81]. The combined OR of people who had a parent who smokes, who were current smokers was 2.33 (CI = 2.23–2.45; 13 282 participants) compared to people who were current smokers whose parents did not smoke. Heterogeneity was low (I^2^ = 0%), and there was no sufficient data to assess publication bias (Figure 4).

## DISCUSSION

This systematic review was conducted to fill a gap in literature by consolidating empirical evidence from available studies in Africa from 2000 to 2020 on key factors associated with adolescent substance use. An accurate understanding of the drivers of adolescent substance use is vitally important for the prevention and treatment of harmful drug and alcohol use as outlined in the SDG (SDG = 3.5). We conducted this systematic review using explicit methods aimed at minimising bias and reliably inform understanding of factors associated with substance use among adolescents within the context of Africa, aiding intervention development by relevant stakeholders. The factors associated with adolescent substance use identified in this review were clustered as individual, family, socio‐economic and environmental and non‐familial social networks. These clusters and the specific factors are discussed below.

In this review, several individual level factors were identified. A total of 60% of the studies reviewed reported gender as a significant factor associated with adolescent substance use. For both the narrative synthesis and meta‐analysis, being male was a significant factor. Specifically, the meta‐analysis showed that being male had an impact on smoking initiation and likelihood to be a current smoker. A total of 43% of studies reported age as a factor associated with substance abuse, particularly older adolescents were at higher odds of using substances. This finding corroborates with other reviews on factors and patterns of adolescent substance use conducted in other regions across the globe [82, 83, 84]. These findings show that prevention strategies should be gender‐sensitive and age appropriate. Particularly with age it highlights the importance of addressing the issue of substance use during adolescence to prevent future negative impacts. In the case of gender, results infer that toxic masculinity plays a significant role in substance use in Africa [85]. Tailored programmes for male adolescents to tease out issues that draw them toward specific substances, including addressing cultural norms around masculinity and substance use is important. A total of 30% of reviewed studies found that mental health problems like depression, anxiety and suicide ideation were associated with substance use among adolescents. In addition, hazardous or concomitant drug and alcohol use were the most common pattern for substance reported in 36.5% included studies in this review. Similarly, a recent systematic review of adolescent drug abuse also found having mood disorders was associated with concomitant substance use [86].

The rest of the factors associated with adolescent substance use reflected external social, community and/or environmental factors. For example, results from the meta‐analysis conducted as part of this systematic review showed that having a friend who smokes was the biggest factor that influenced smoking initiation. Having a family member who smokes also had an impact on smoking initiation. For current smokers, having at least one family member that smokes was again a significant factor. These findings were corroborated by the narrative synthesis where 40% of the included articles reported living with a family member who uses a substance as one of the associated factors. Lacking parental supervision was also highlighted in 19% of the studies as an additional risk factor within the family context. This suggests that alongside personal characteristics like gender and age, substance use is also implicated in social contexts, including familial and non‐familial relationships. It is well known that supportive relationships with caring family, partners and friends who do not use substances themselves are helpful in abstaining and maintaining sobriety [87]. It is, therefore, not surprising that surrounding oneself with users increases an individual's likelihood of substance use.

The bulk of our data was from self‐reported surveys from school‐based adolescents, collected at single time points. It is, therefore, difficult to assess changes over time. Surveys like the GSHS and GYTS are relatively low‐cost, self‐administered questionnaires used to obtain data on young people's health behaviour and protective factors related to the leading causes of morbidity and mortality among children and adults worldwide. They have standardised methodology for constructing the sampling frame, selecting schools and classes, preparing questionnaires, following consistent field procedures and using consistent data management procedures for data processing and analysis. The overuse of such low‐cost standardised surveys may again be a reflection of limited funding for most researchers in Africa, which limits ability to conduct studies with more complex study designs (e.g. clinical trials) with more time intensive and costly methodological approaches [88]. However, most of these surveys contain items with Likert style preassigned responses instead of free text responses, therefore, the counts may not reflect the weight of indicated risk factors. The way some questions on substance use behaviours are asked may influence how participants respond and may be subject to social desirability bias or non‐response bias [89]. A total of 60% of the review data came from school going students and we were unable to pick up differences from the few studies with more unique populations like children who were homeless or living on the streets [32, 33]. We were also unable to assess culture within the different countries across included studies and how these influence unique types of substance use behaviours. Previous literature alludes that this is heterogeneous with certain substances being more popular in specific geographical regions on the continent [8]. A future ethnographic systematic review focused on qualitative studies is required to more suitably explore perceptions, experiences and behaviours related to adolescent substance use on the continent in the context of variable culture [90].

On a broader level, there was an imbalance in the number of included studies per region with Southern Africa having significantly more studies, whereas North Africa had the least. On a country level, most included studies were conducted in South Africa. There was equal number of studies between West and East African regions. This imbalance may be a reflection of the different research infrastructure across regions on the continent. These differences in infrastructure feed into issues such as access to research funding, availability of academic researchers and research dissemination including publication bias. Indeed, most of the included studies for this review did not have or declare research funding, with many relying on readily existing data to conduct secondary analyses on. It is well known that researchers based outside European and American regions do not work in enabling environments where funding and support required to thrive are easily accessible. There is also a general lack of investment in data storage infrastructure in most African countries. A needs assessment of research institutions across seven countries in Africa reported underinvestment in both physical and digital research infrastructure, a low number of researchers and incomplete, under‐resourced national institutional frameworks [88]. Such critical gaps typically result in differences in research production, which can limit research dissemination, international visibility and national influence on domestic economic, social or environmental issues. In the case of our review, we are conscious that limited availability of empirical data does not mean absence of adolescent substance use issues within the various societies. This is a bigger challenge for those working in areas related to mental health research, which is chronically underfunded [91].

### Strengths and limitations

We used stringent systematic methodology throughout the review process as outlined in our protocol [18] using PRISMA guidance for reporting of systematic review to facilitate transparent reporting of why this review was done, how and key findings [92]. Several factors were assessed with a bottom‐up approach to our coding and thematic analysis, not limited to one aspect or level (Appendix S1). In addition, the search strategy was not restricted to a specific study design. Both quantitative and qualitative studies were included to ensure a broad scope of data was captured for the review. We did not restrict our search strategy in terms of language, which meant we came across studies written in English, French and German during the initial screening process, having multilingual team members facilitated this process.

Nonetheless, this review has several limitations. First, our results should only be considered in the context of the databases we used to search the literature, which might not be comprehensive. Second is the language of articles published. Although we chose not to exclude based on language, most of the literature we came across was in English with few others in French and German. The third limitation is around publication bias, where documents not published on‐line as well as reports not found in peer reviewed journals were not considered. Fourth, our choice to exclude few articles with cohorts that had some age groups outside the chosen definition of adolescence (e.g. cohorts age 16 to 24 years) may underestimate some statistical outcomes. Fifth, the review included observational studies, therefore, we are unable to determine causality and can only infer on the association between reported factors and substance use. Moreover, the quality of most included studies was low, therefore, findings may be subject to some methodological flaws of the underlying articles. For instance, non‐standardisation of constructs such as age, substance use type or substance use outcomes were prominent. Some results were presented separately for age or gender groups to adjust for confounders, but simple univariate analysis was removed and, therefore, could not be combined easily. This heterogeneity in terms of type and length of substance use, definitions of study outcomes and predictors across the included articles meant that there were very few articles with data that could be suitably combined for meta‐analyses.

## CONCLUSION

This study presents multiple factors that operate on individual, family and societal levels that have significant influence on use of substances during adolescence. Interestingly, key factors of adolescent substance use, namely being male, an older adolescent and exposed to peer substance use, identified in this review focused on African studies are similarly observed in European and North American studies [86]. This suggests a potential for cross cultural collaborations to develop targeted interventions for adolescent groups from both high and low resource settings across the globe. Successful substance abuse prevention programs need to account for the complex interaction of these multiple factors across all levels of domains. We suggest appropriate guidance and counselling regarding the harmful use alcohol and drugs to increase knowledge about their effects on one's health and general functioning, wider family distress and the potential loss of future productivity that places an economic burden on the wider community. This should target local communities with a focus on structures and systems where young people and their families naturally preside. More research is needed that focuses on how being male intersects with traditional ideologies, societal expectations and impacts on how men relate with substance use from a young age in Africa, especially the use of alcohol and drugs as coping mechanisms [85, 93]. We also recommend development of holistic policies that address the strong influence of family and peers on adolescent substance use in African countries observed in reviewed studies.

## AUTHOR CONTRIBUTIONS

**Sandra Jumbe:** Conceptualization (lead); data curation (lead); formal analysis (lead); funding acquisition (lead); investigation (equal); methodology (equal); project administration (equal); resources (lead); supervision (lead); validation (equal); visualization (lead); writing—original draft (lead); writing—review and editing (equal). **Tony Mwenda Kamninga:** Conceptualization (equal); data curation (supporting); formal analysis (supporting); investigation (equal); methodology (equal); resources (equal); validation (equal); visualization (supporting); writing—review and editing (equal). **Ukwuori‐Gisela Kalu:** Conceptualization (equal); data curation (equal); formal analysis (supporting); investigation (equal); methodology (equal); validation (supporting); visualization (supporting); writing—review and editing (supporting). **Joel Nyali:** Data curation (equal); formal analysis (equal); investigation (equal); project administration (equal); validation (supporting); writing—original draft (supporting). **Lara Saleh:** Data curation (supporting); formal analysis (supporting); investigation (supporting); validation (supporting); writing—original draft (supporting); writing—review and editing (supporting). **Chris Newby:** Formal analysis (equal); investigation (equal); methodology (supporting); validation (supporting); writing—original draft (supporting); writing—review and editing (equal). **Joel Msafiri Francis:** Formal analysis (supporting); investigation (supporting); methodology (supporting); supervision (supporting); validation (equal); visualization (equal); writing—review and editing (equal).

## DECLARATIONS OF INTEREST

None.

## CLINICAL TRIAL REGISTRATION

PROSPERO CRD42020190158.

## Supporting information


**Appendix S1:** Coding of factors of adolescent substance use in Africa to determinants.
**Appendix S2:** Predictors of adolescent alcohol use mentioned across included studies.
**Appendix S3:** Predictors of adolescent smoking mentioned across included studies.

**Table S1:** Key characteristics and quality ratings of included studies.

## Data Availability

The data that support the findings of this study are available from the corresponding author upon reasonable request.

## Footnotes

1
https://www.who.int/europe/about-us/our-work/sustainable-development-goals/targets-of-sustainable-development-goal-3
