# Supplementary material for: A systematic review and meta‐analysis of factors associated with adolescent substance use in Africa, 2000 to 2020
Source: Addiction. 2025 Feb 27;120(6):1127–42. doi: 10.1111/add.70023 (PMC12046489; doi:10.1111/add.70023)
Supplement: Supplementary file 1 — Appendix S1: Coding of factors of adolescent substance use in Africa to determinants. Appendix S2: Predictors of adolescent alcohol use mentioned across included studies. Appendix S3: Predictors of adolescent smoking mentioned across included studies. [file ADD-120-1127-s002.pptx]

## Slide 1
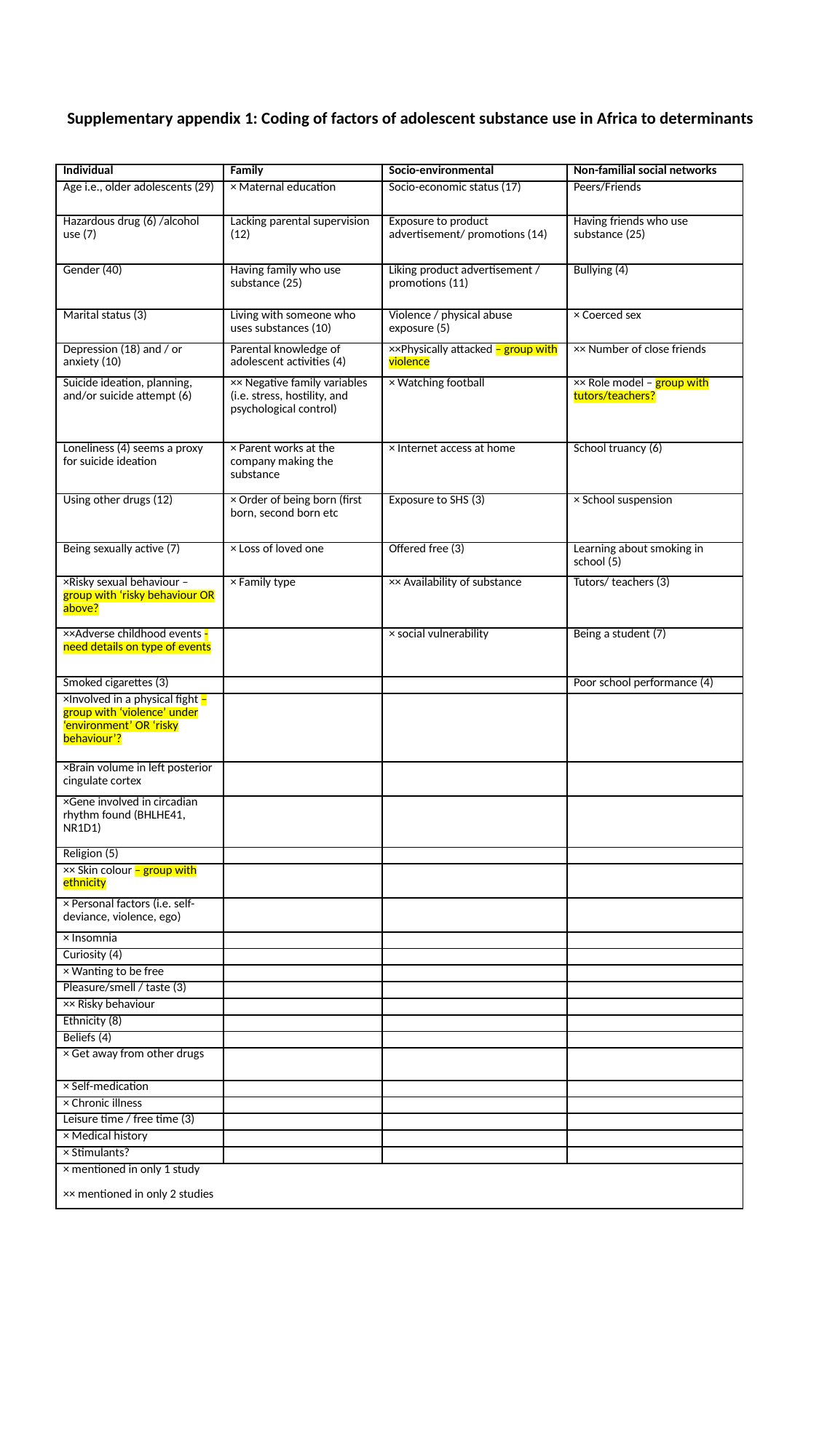

Supplementary appendix 1: Coding of factors of adolescent substance use in Africa to determinants
| Individual | Family | Socio-environmental | Non-familial social networks |
| --- | --- | --- | --- |
| Age i.e., older adolescents (29) | × Maternal education | Socio-economic status (17) | Peers/Friends |
| Hazardous drug (6) /alcohol use (7) | Lacking parental supervision (12) | Exposure to product advertisement/ promotions (14) | Having friends who use substance (25) |
| Gender (40) | Having family who use substance (25) | Liking product advertisement / promotions (11) | Bullying (4) |
| Marital status (3) | Living with someone who uses substances (10) | Violence / physical abuse exposure (5) | × Coerced sex |
| Depression (18) and / or anxiety (10) | Parental knowledge of adolescent activities (4) | ××Physically attacked – group with violence | ×× Number of close friends |
| Suicide ideation, planning, and/or suicide attempt (6) | ×× Negative family variables (i.e. stress, hostility, and psychological control) | × Watching football | ×× Role model – group with tutors/teachers? |
| Loneliness (4) seems a proxy for suicide ideation | × Parent works at the company making the substance | × Internet access at home | School truancy (6) |
| Using other drugs (12) | × Order of being born (first born, second born etc | Exposure to SHS (3) | × School suspension |
| Being sexually active (7) | × Loss of loved one | Offered free (3) | Learning about smoking in school (5) |
| ×Risky sexual behaviour – group with ‘risky behaviour OR above? | × Family type | ×× Availability of substance | Tutors/ teachers (3) |
| ××Adverse childhood events - need details on type of events | | × social vulnerability | Being a student (7) |
| Smoked cigarettes (3) | | | Poor school performance (4) |
| ×Involved in a physical fight – group with ‘violence’ under ‘environment’ OR ‘risky behaviour’? | | | |
| ×Brain volume in left posterior cingulate cortex | | | |
| ×Gene involved in circadian rhythm found (BHLHE41, NR1D1) | | | |
| Religion (5) | | | |
| ×× Skin colour – group with ethnicity | | | |
| × Personal factors (i.e. self-deviance, violence, ego) | | | |
| × Insomnia | | | |
| Curiosity (4) | | | |
| × Wanting to be free | | | |
| Pleasure/smell / taste (3) | | | |
| ×× Risky behaviour | | | |
| Ethnicity (8) | | | |
| Beliefs (4) | | | |
| × Get away from other drugs | | | |
| × Self-medication | | | |
| × Chronic illness | | | |
| Leisure time / free time (3) | | | |
| × Medical history | | | |
| × Stimulants? | | | |
| × mentioned in only 1 study ×× mentioned in only 2 studies | | | |

## Slide 2
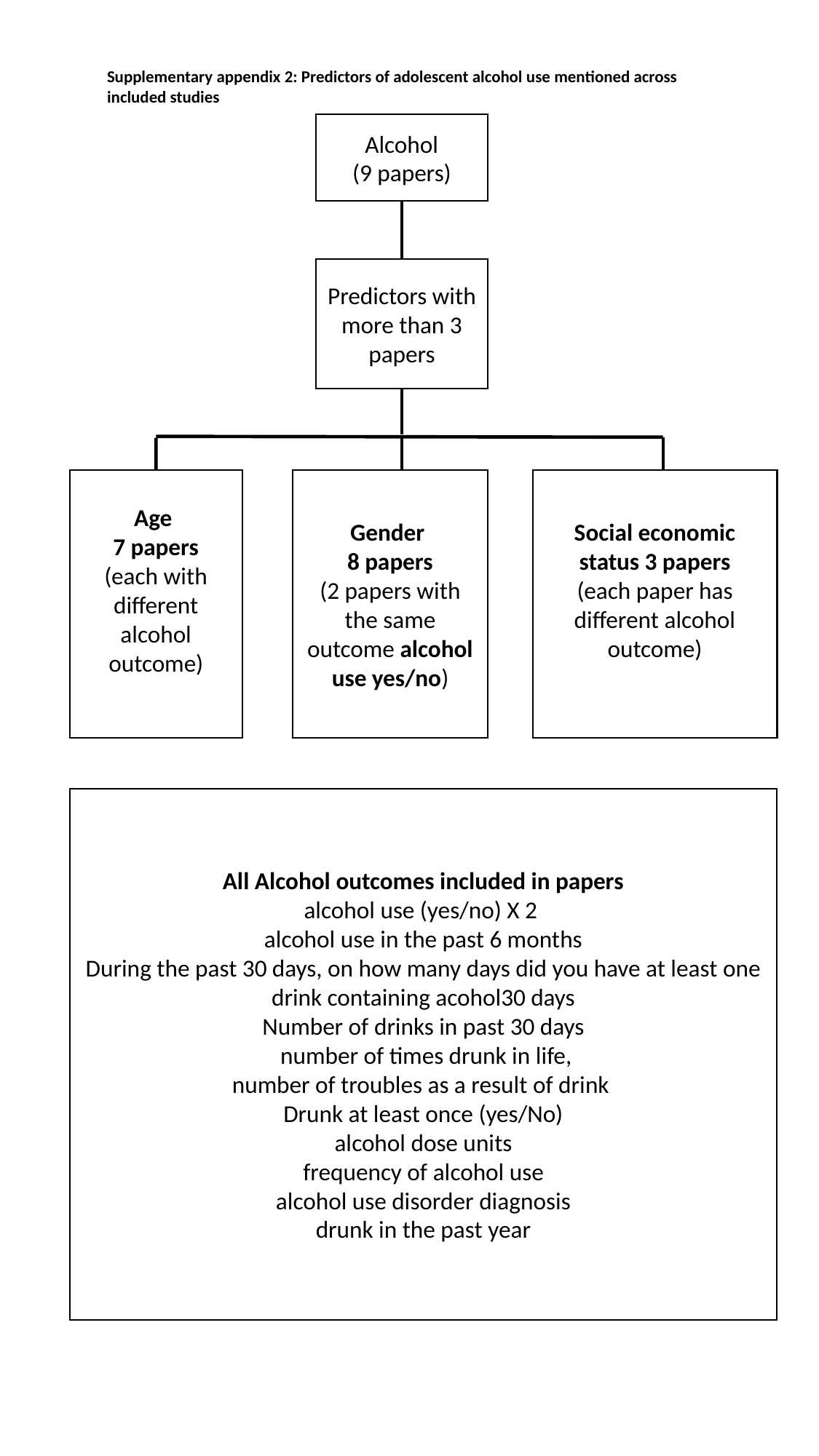

Supplementary appendix 2: Predictors of adolescent alcohol use mentioned across included studies
Alcohol
(9 papers)
Predictors with more than 3 papers
Gender
8 papers
(2 papers with the same outcome alcohol use yes/no)
Social economic status 3 papers
(each paper has different alcohol outcome)
Age
7 papers
(each with different alcohol outcome)
All Alcohol outcomes included in papers
alcohol use (yes/no) X 2
alcohol use in the past 6 months
During the past 30 days, on how many days did you have at least one drink containing acohol30 days
Number of drinks in past 30 days
 number of times drunk in life,
number of troubles as a result of drink
Drunk at least once (yes/No)
alcohol dose units
frequency of alcohol use
alcohol use disorder diagnosis
drunk in the past year

## Slide 3
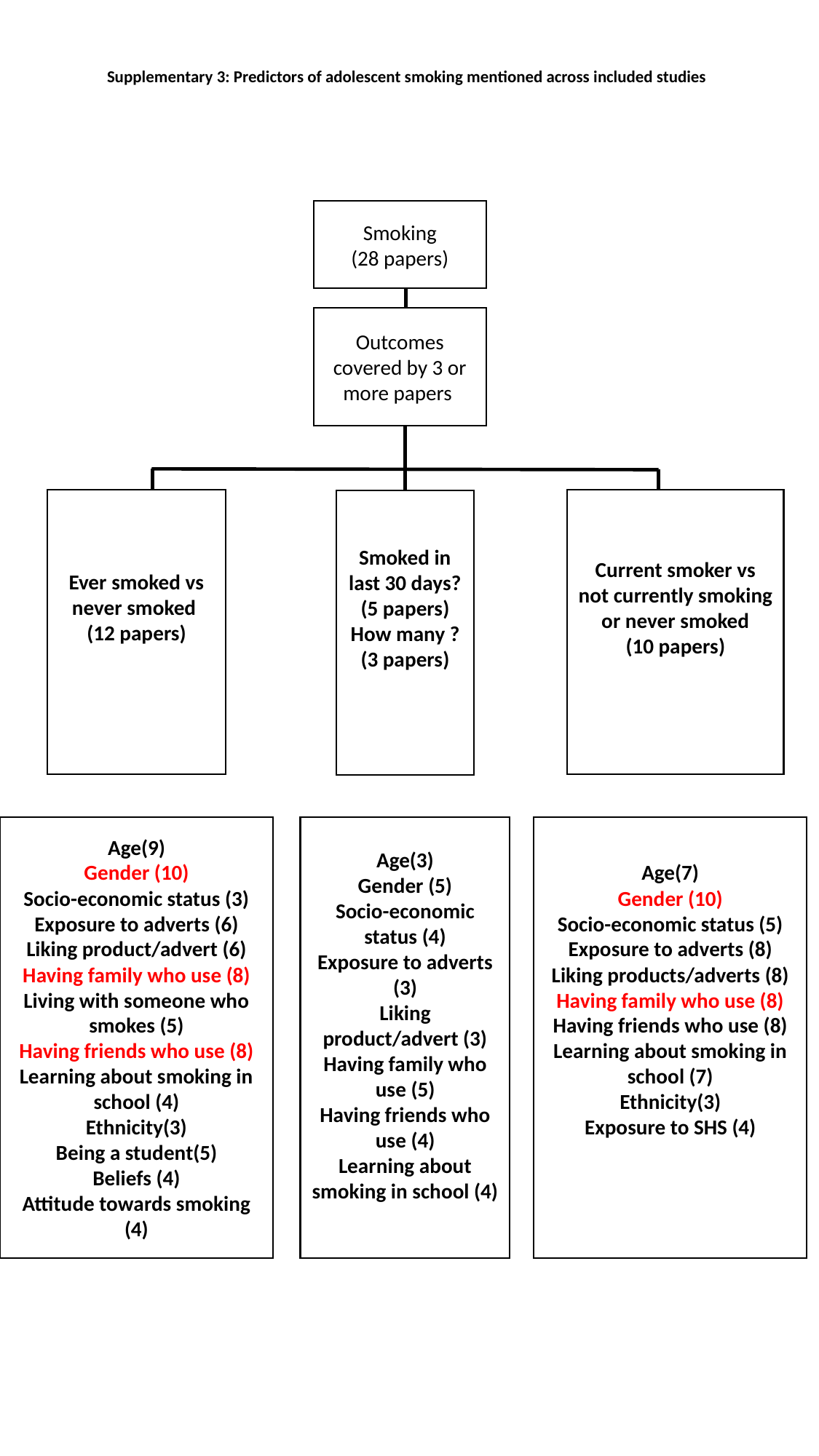

Supplementary 3: Predictors of adolescent smoking mentioned across included studies
Smoking
(28 papers)
Outcomes covered by 3 or more papers
Ever smoked vs never smoked
(12 papers)
Current smoker vs not currently smoking or never smoked
(10 papers)
Smoked in last 30 days?
(5 papers)
How many ?
(3 papers)
Age(9)
Gender (10)
Socio-economic status (3)
Exposure to adverts (6)
Liking product/advert (6)
Having family who use (8)
Living with someone who smokes (5)
Having friends who use (8)
Learning about smoking in school (4)
Ethnicity(3)
Being a student(5)
Beliefs (4)
Attitude towards smoking (4)
Age(3)
Gender (5)
Socio-economic status (4)
Exposure to adverts (3)
Liking product/advert (3)
Having family who use (5)
Having friends who use (4)
Learning about smoking in school (4)
Age(7)
Gender (10)
Socio-economic status (5)
Exposure to adverts (8)
Liking products/adverts (8)
Having family who use (8)
Having friends who use (8)
Learning about smoking in school (7)
Ethnicity(3)
Exposure to SHS (4)
