# Supplementary material for: A systematic review and meta‐analysis of factors associated with adolescent substance use in Africa, 2000 to 2020
Source: Addiction. 2025 Feb 27;120(6):1127–42. doi: 10.1111/add.70023 (PMC12046489; doi:10.1111/add.70023)
Supplement: Supplementary file 2 — Table S1: Key characteristics and quality ratings of included studies. [file ADD-120-1127-s001.docx]

| **Supplementary table 1: Key characteristics and quality ratings of included studies** | | | | | | | | | |
| --- | --- | --- | --- | --- | --- | --- | --- | --- | --- |
| **No** | **Author & year** | **Title** | **Country** | **Sample size** | **Sample**  **Details** | **Age range** | **Substance type** | **Factors associated with substance use** | **Quality rating** |
| 1 | Ramsoomar et al 2013 | Alcohol use in early and late adolescence among the birth to twenty cohort in Soweto, South Africa | South Africa | 3356 | early (13 years) and late (18 years) adolescence in Soweto, South Africa | 13-18 | Alcohol | Age (older adolescents), Gender- males+, maternal marital status-married mothers less use of alcohol by children , maternal education- educates mothers less alcohol consumption, social economic status- poverty and alcohol use | Moderate |
| 2 | Morojele et al 2018 | Alcohol marketing and adolescent alcohol consumption: Results from the International Alcohol Control study (South Africa) | South Africa | 869 | 408 males and 461 females aged 16 and 17 years,  869 adolescents, 71 (10.6%, 95% CI 5.9 - 18.3 - weighted) of whom reported alcohol consumption in the past 6 months | 16-17 | Alcohol | Exposure to product advertisement/ promotion, Depression aka. low mood, sad, hopeless. | Moderate |
| 3 | Siziya et al 2009 | Alcohol use among school-going adolescents in Harare, Zimbabwe: results from the 2003 Global School-Based Health Survey | Zimbabwe | 1997 | Adolescent students in Harare, Zimbabwe. No specific age range given but about a third (31.3%) of students were of age 15 years, | 13-16 | Alcohol | Depression aka. low mood, sad, hopeless.  Suicide ideation, planning, and/or suicide attempt, lack of parental supervision, Bullying, Truancy, Being sexually active-, Smoked cigarettes, | Weak |
| 4 | Oppong Asante & Kugbey, 2019 | Alcohol use by school-going adolescents in Ghana: Prevalence and correlates | Ghana | 1984 | The 2012 version of the Ghanaian Global School-based Student Health Survey on adolescents aged 11-19 years old (N = 1984) was used | 11-19 | Alcohol | Anxiety a.k.a worries, fears, Suicide ideation, planning, and/or suicide attempt, loneliness, bullying, truancy, smoked cigarettes, Involved in a physical fight, Parental knowledge of adolescent activities- more checking reduces risk, Physical attacked, | Moderate |
| 5 | Parry et al 2004 | Brief report: Social and neighbourhood correlates of adolescent drunkenness: a pilot study in Cape Town, South Africa | South Africa | 90 | 90 adolescents aged 11–17 years were selected from nine distinct communities using a multi stage, cluster sampling strategy. In the first stage we stratified communities by race with the predominant race group1 in a community used to classify the community’s race group. We randomly selected one lower-, one middle- and one higher-income community within each race stratum. | 11-17 | Alcohol | Age (older adolescents), Having friends who use substance-peer pressure | Strong |
| 6 | Dalvie et al 2018 | Genetic variation within GRIN2B in adolescents with alcohol use disorder may be associated with larger left posterior cingulate cortex volume | South Africa | 116 | The study participants were predominantly Afrikaans, followed by English speaking and the median number of years of education was 8.0 years for both groups (HC and AUD). | 11-17 | Alcohol | Brain volume in left posterior cingulate cortex- | Strong |
| 7 | Page & Hall 2009 | Psychosocial Distress and Alcohol Use as Factors in Adolescent Sexual Behaviour Among Sub-Saharan African Adolescents | Uganda, Kenya, Zimbabwe, Namibia, Zambia and Botswana | 22,949 | 22,949 adolescents from Botswana, Kenya, Namibia, Uganda, Zambia, and Zimbabwe who participated in 2003 or 2004 GSHS | 11-16 | Alcohol | Depression aka. low mood, sad, hopeless. Being sexually active, | Weak |
| 8 | Dalvie et al 2016 | Possible involvement of the circadian pathway in alcohol use disorder in a South African adolescent cohort | South Africa | 80 | Our cohort consisted of 80 adolescents with an AUD diagnosis and 80 matched controls of mixed ancestry ethnicity. In total, there were 47 females and 33 males in both the AUD and HC groups. | 14.94 years (IQR 15.53-14.47) AUD group. 14.92 (IQR 15.33-14.36) HC group median age | Alcohol | Gene involved in circadian rhythm found (BHLHE41, NR1D1) | Weak |
| 9 | Kabiru et al 2010 | Self-reported drunkenness among adolescents in four sub-Saharan African countries: associations with adverse childhood experiences | Burkina Faso, Ghana, Malawi, and Uganda | 9819 | Data from 9,819 adolescents aged 12-19 years (mean age 15.0, SD 2.21) from Burkina Faso Ghana, Malawi, and Uganda. | 12-19 | Alcohol | Gender- males more than females , Having family who use substance, Living with someone who uses substances, Adverse childhood events, Violence / physical abuse exposure, coerced sex | Weak |
| 10 | Agbo et al 2009 | Cigarette smoking habits among adolescents in northeast Nigeria | Nigeria | 171 | A cross-sectional study was carried out in Yola south Local Government Area (LGA) of Adamawa state, northeast  Nigeria. The target population was adolescents aged 12  to 17 years. | 12-17 | Tobacco | Exposure to product advertisement/ promotion, Having family who use substance, Living with someone who uses substances, Having friends who use substance | Moderate |
| 11 | Ouédraogo et al 2015 | Prevalence of smoking among 741 High school students from Dakar. Carbon monoxide measurement | Senegal | 741 | 741 High school students from Dakar | 11-18 | Tobacco | Having family who use substance, Living with someone who uses substances, Having friends who use substance, Curiosity, Wanting to be free | Moderate |
| 12 | Monyeki et al 2013 | Advertisement and knowledge of tobacco products among Ellisras rural children aged 11 to 18 years: Ellisras Longitudinal study | South Africa | 1654 | Ellisras rural children aged between 11 to 18 years who are part of the Ellisras Longitudinal Study (ELS | 11-18 | Tobacco | Exposure to product advertisement/ promotions, Liking product advertisement/ promotions | Moderate |
| 13 | Veeranki et al 2017 | Age of smoking initiation among adolescents in Africa | Cape Verde, Cote d'Ivoire, Ghana, Guinea, Mali, Mauritania, Niger, Senegal, and Togo | 16519 | The study data were obtained from the GYTSs conducted in nine West African countries (Cape Verde, Cote d’Ivoire, Ghana, Guinea, Mali, Mauritania, Niger, Senegal,  and Togo) during 2006–2009. Only countries with  surveys that collected nationally representative tobacco related  information on school-going adolescents were included. | 13-15 | Tobacco | Age (older adolescents at risk), gender- more males at risk, Exposure to product advertisement/ promotions, Liking product advertisement/ promotions, Having family who use substance, Having friends who use substance, Being a student- influence of the environment | Weak |
| 14 | Muula 2007 | Prevalence and determinants of cigarette smoking among adolescents in Blantyre City, Malawi | Malawi | 1308 | School-going adolescents in Blantyre in 2001 who completed the Global Youth Tobacco Survey. | 11-17 | Tobacco | Age -older adolescents at risk, Gender- males at risk, Exposure to product advertisement/ promotions, Liking product advertisement/ promotions, Having family who use substance, Living with someone who uses substances, Having friends who use substance, Learning about smoking in school- reduces risk of substance use | Weak |
| 15 | Muula & Siziya 2007 | Prevalence and determinants of ever smoked cigarettes among school-going adolescents in Lusaka, Zambia | Zambia | 2175 | 2175 school-going adolescents in Lusaka, Zambia. Majority of students within the 13 to 15 years age range were randomly chosen. The selected students were in Grades 7, 8 and 9 | age range or mean not reported | Tobacco | Age (Older adolescent, Gender- males at risk, Exposure to product advertisement/ promotions, Liking product advertisement/ promotions, Having family who use substance, Living with someone who uses substances, Having friends who use substance, Learning about smoking in school- reduces the risk, pleasure/smell/taste, No reason | Moderate |
| 16 | Kaduri et al 2008 | Smokeless tobacco use among adolescents in Ilala Municipality, Tanzania | Tanzania | 1010 | 1011 students randomly selected; boys (mean age= 14.5 years) accounted for 50.7% and girls (mean age= 13.6 years) 49.3%. | 10-18 | Tobacco | Gender-males, Exposure to product advertisement/ promotions, Liking product advertisement/ promotions, Being a student- influence of environment and friends | Weak |
| 17 | Rudatsikira et al 2007 | Prevalence and determinants of adolescent tobacco smoking in Addis Ababa, Ethiopia | Ethiopia | 1868 | 11-17 year old school going adolescents in Addis Ababa | 11-17 | Tobacco | Gender-males, Exposure to product advertisement/ promotions, Liking product advertisement/ promotions, Having family who use substance, Living with someone who uses substances, Having friends who use substance, | Strong |
| 18 | Muula & Mpabulungi 2007 | Cigarette smoking prevalence among school-going adolescents in two African capital cities: Kampala Uganda and Lilongwe Malawi | Uganda and Malawi | 3115 | 1820 students participated in Lilongwe, Malawi of whom 869 (47.8%) were males, 856 (47.0%) were females and 95 (5.2%) were missing. In Kampala, 2789 students participated of whom 1295 (46.4%) were males, 1323 (47.4%) females and 171 (6.1%) were missing. For Lilongwe students were from standard 7 to Form 1 while in Kampala students were from senior 1 to 3 (form 1 to 3). The median age in both cities was 14 years. | 13-15 | Tobacco | Age (older adolescents), Exposure to product advertisement/ promotions, Liking product advertisement/ promotions. | Strong |
| 19 | Panday et al 2007 | Determinants of smoking among adolescents in the Southern Cape-Karoo region, South Africa | South Africa | 3869 | In 2002, a cross-sectional survey was administered to grade 9–11 students (14–16 years of age) in the Southern Cape-Karoo Region, Western Cape Province. Forty-two public schools were eligible to participate in the study. On the basis of previous research (Panday et al., 2003), school selection was stratified by ethnicity in accordance with the school’s previous race classification, namely, Black African (six schools), Coloured (17 schools) or White (19 schools). A total of 23 schools were selected to participate in the study, consisting of all six Black African schools, eight randomly selected Coloured schools and nine randomly selected White schools. | 14-16 | Tobacco | Gender- males at more risk, Depression aka. low mood, sad, hopeless, Having family who use substance, Risky behaviour, Ethnicity - coloured students, whites students then black Africana at higher chance of smoking respectively. | Strong |
| 20 | Panday et al 2005 | Determinants of smoking cessation among adolescents in South Africa | South Africa | 1267 | A cross-sectional study was conducted in 2002 in the Southern Cape-Karoo Region, Western Cape Province. Grade 9–11 students (general age range of 14–16 years) completed a self-administered questionnaire. Forty-two public schools were eligible to participate in the study. School selection was stratified by ethnicity in accordance with the school’s previous race classification under the apartheid government, i.e. Black African (six schools), Colored (17 schools) or White (19 schools). | 14-16 | Tobacco | Age (older adolescents), Gender-males at risk, Depression aka. low mood, sad, hopeless, Having family who use substance, Having friends who use substance, Learning about smoking in school, Risky behavior, Ethnicity-, Tutors/ teachers- role models hence influence | Strong |
| 21 | Odukoya et al 2013 | Determinants of smoking initiation and susceptibility to future smoking among school-going adolescents in Lagos State, Nigeria | Nigeria | 1031 | This study was conducted in Lagos State, the commercial capital of Nigeria, a country with an estimated population of 162 million people as at 2011, of which the youth are estimated to be over 30 percent. (Population Reference Bureau, 2009; World Bank, 2012) There are twenty local government areas (LGA) in the state, sixteen of which are classified as urban. There were 609 public schools, and 421 private schools registered in Lagos state as at the time of the survey. | 10-21 | Tobacco | Age (older adolescents), Gender- males at more, having family who use substance, Having friends who use substance, Being a student-environmental influence | Strong |
| 22 | Fernander et al 2006 | Gender differences in depression and smoking among youth in Cape Town, South Africa | South Africa | 946 | Data for this study were derived from the 1997 South African Community Epidemiology Network on Drug Use (SACENDU) School survey. A stratified sampling procedure utilizing postal zip codes was used to select students at nonprivate high schools in Cape Town. The number of schools selected was proportional to the total number of students in all the schools in the geographic stratum. The probability of selection of a school was proportional to the number of students in the school. Within each selected school (n539), two classes in each of two grades (8 and 11) were randomly chosen and 40 students were randomly selected. A maximum of five additional students per grade were selected as replacements for absentees, drop-outs, and transferees | Not explicitly said but average of 17 years. Even though the age range is not explicitly described, the study targets students in grades 8 to 11 which are typically 13- to 16-year-olds in South Africa | Tobacco | Age (older adolescents), Gender- males use more than females, Depression aka. low mood, sad, hopeless | Strong |
| 23 | Islam & Johnson, 2005 | Influence of known psychosocial smoking risk factors on Egyptian adolescents' cigarette smoking behavior | Egypt | 1930 | The study was conducted in the city of Alexandria, Egypt. Alexandria is Egypt’s second largest city and main seaport. It has a population of _4 million people, primarily comprising indigenous ethnic Egyptian groups (Ashmawy et al., 2000). Schools were stratified based on school type (public/private) and student gender. Two schools from within each stratum were randomly selected, for a total of eight schools. Three classes per grade per school were randomly selected to participate in the study. Data were obtained from all actively consenting students in grades 7, 9 and 12. | 13-19 | Tobacco | Gender- males use more than females, Having family who use substance, Living with someone who uses substances, Having friends who use substance, Being a student, beliefs- smoking has positive outcomes | Strong |
| 24 | Talley et al 2017 | Multilevel analysis of school anti-smoking education and current cigarette use among South African students | South Africa | 3068 | Data for the current study are from the full GYTS conducted in SA during 2011; the full study included a total of 10,833 students in grades 8-11. For the current study, students aged 13-15 years were included as outlined in the GYTS protocol (n=3947). In addition to the age range restriction, the study examined only cases with complete information on covariates of interests, which resulted in an analytic sample of n=3068. To evaluate the potential impact of missing data, we compared pairwise present associations between each predictor and the outcome for all students aged 13-15 years (n=3947) with the final analytic sample (n=3068). | 13-15 | Tobacco | Age-older adolescents, Gender (males use more than females), Exposure to product advertisement/ promotions, Liking product advertisement/ promotions, Having family who use substance, Having friends who use substance, Learning about smoking in school- reduces the influence to school, Exposure to SHS, Offered free, Role models-influence | Moderate |
| 25 | Peltzer, 2011 | Early smoking initiation and associated factors among in-school male and female adolescents in seven African countries | Botswana, Kenya, Namibia, Senegal, Tanzania, Uganda and Zimbabwe | 17725 | This study involved secondary analysis of existing data from the Global school-based health survey (GSHS) from seven African countries (Botswana, Kenya, Namibia, Senegal, Tanzania, Uganda and Zimbabwe, Swaziland and Zambia did not collect information on smoking). All African countries from which GSHS datasets were publicly available were included in the analysis. From all but two cof the countries national samples were included, while from Tanzania it was only the Dar es Salaam region and from Zimbabwe three areas: Harare, Bulawayo and Manicaland. | 13-15 | Tobacco | Depression aka. low mood, sad, hopeless, Anxiety a.k.a. worries, fears, Suicide ideation, planning, and/or suicide attempt, Lacking parental supervision, truancy, Having family who use substance, Being sexually active, Physical violence/abuse exposure, Leisure/free time-time wasting activities activity, Availability of substance | Moderate |
| 26 | Muula et al 2008 | Prevalence and correlates of cigarette smoking among adolescents in Malawi: results from the Global Youth Tobacco Survey 2005 | Malawi | 5135 | A nationally representative sample of 5135 students (in school adolescents) aged 12 to 15 years in Malawi. | 12-15 | Tobacco | Gender (males at more risk to use substance, Socio-economic status-pocket money , Exposure to product advertisement/ promotions, Liking product advertisement/ promotions, Having family who use substance, Having friends who use substance, Curiosity-wanting to know the feel, Beliefs- religion, belief smoking makes one lose weight, Offered free | Moderate |
| 27 | Kapito-Tembo et al 2011 | Smoking among in-school adolescents in Dares Salaam, Tanzania: results from the Global Youth Tobacco Survey | Tanzania | 1947 | The Dare es Salaam (Tanzania) GYTS conducted in 2003 was a cross-sectional study, aimed to recruit in-school adolescents 13 to 15 years old to estimate prevalence of tobacco use. Data were also collected on socio-demographic characteristics, exposure to media, parental smoking, peer smoking and exposure to support that may assist the teenager to stop smoking or not start smoking. | 13-15 | Tobacco | Age- older adolescents, Gender- males at more risk, Socioeconomic status-having pocket money, Curiosity | Strong |
| 28 | Brook et al 2009 | The Relationship Between Receptivity to Media Models of Smoking and Nicotine Dependence Among South African Adolescents | South Africa | 731 | The sample for this study consisted of 731 adolescents (53% female) residing in Johannesburg, South Africa. The adolescents were 35% “Black African” (N=259), 30% “Coloured” (a South African term for people of mixed ancestry, N=222), 27% “Indian” (N=194), and 8% “White” (N=56). Their ages ranged from 12 to 17 years with a mean age of 14.55 years (SD=1.68). | 12-17 | Tobacco | Gender –males smoke more, Exposure to product advertisement/ promotions, Ethnicity- whites smoke more than blacks, Beliefs-attitudes and beliefs influenced by smoking models | Moderate |
| 29 | Brook et al 2005 | Predictors of Cigarette Use Among South African Adolescents | South Africa | 1468 | This study examines a sample of 1468 adolescents, drawn from two cities in South Africa, Durban and Cape Town in 2001–2002. The sample is 45% male and 55% female. The subjects are divided into four ethnic groups: 660 (45%) are African/Black (mainly Zulu and Xhosa); 204 (14%) are Indian; 289 (20%) are White; 311 (21%) are Coloured (a South African term for people of mixed ancestry). The adolescents’ range in age from 12 to 17 years, with a mean of 14.7 years (SD=1.9). | 12-17 | Tobacco | Age-older adolescents, Gender- males at more risk of use, Depression aka. low mood, sad, hopeless, Anxiety a.k.a worries, fears, Anxiety a.k.a. worries, fears, Loneliness, Lacking parental supervision, Bullying, Truancy, Having family who use substance, Having friends who use substance, Ethnicity- 24.8% of “Coloured” sample were current smokers, 10.7% of the black sample, 17.6% of the white sample, and 9.8% of the Indian sample. | Strong |
| 30 | Brook et al 2006 | Personal, Interpersonal, and Cultural Predictors of Stages of Cigarette Smoking Among Adolescents in Johannesburg, South Africa | South Africa | 731 | We interviewed 731 adolescents, 47% male and 53% female, drawn from communities in and around Johannesburg, South Africa. They ranged in age from 12–17 years, with a mean age of 14.5 years (SD 1.68). At the time of the interviews 96% of the adolescents reported being in school, and the mean educational level was 8th grade. The ethnic breakdown of the sample was 35.4% black (n = 59), 30.4% ‘‘coloured’’ (a South African term for mixed ancestry; n = 222), 26.5% Indian (n = 194), and 7.7% white (n = 56). | 12-17 | Tobacco | Age-older adolescents, gender-males, ethnicity-whites smoke more than blacks | Strong |
| 31 | Itanyi et al 2020 | Predictors of current tobacco smoking by adolescents in Nigeria: Interaction between school location and socioeconomic status | Nigeria | 4332 | Eligible participants were students in Junior Secondary 2 and 3, and Senior Secondary 1 (i.e. JS2, JS3, SS1) corresponding to 8th, 9th and 10th grade. There were 4332 adolescents out of 4354 respondents, with 2230 and 2102 students from urban and rural schools, respectively. Response rates were 84.4% in urban and 80.6% in rural locations. Participants were 10-19 years old. Sociodemographic characteristics included age group (10-12, 13-15 and 16-19 years) | 10-19 | Tobacco | Gender- males, Socio-economic status- rich parents more risk to child to smoke, Exposure to product advertisement/ promotions, Liking product advertisement/ promotions, Lacking parental supervision, Having family who use substance, Having friends who use substance, Learning about smoking in school- reduces the risk, Being a student, Leisure time/free time | Moderate |
| 32 | Lee et al 2016 | Second hand smoke exposure and susceptibility to initiating cigarette smoking among never-smoking students in selected African countries: Findings from the Global Youth Tobacco Survey | Twenty-nine African countries (Botswana, Burundi, Cape Verde, Chad, Comoros, Congo, Côte d'Ivoire, Equatorial Guinea, Eritrea, Ghana, Guinea, Kenya, Lesotho, Madagascar, Malawi, Mali, Mauritania, Mauritius, Namibia, Niger, Rwanda, Sao Tome and Principe, Senegal, Seychelles, South Africa, Swaziland, Togo, Uganda, and Zambia) | 56967 | GYTS data from 29 countries in the WHO Africa region to explore prevalence of SHS exposure at home and in public places, as well as susceptibility to initiate cigarette smoking among youth who have never smoked in 29 African countries using GYTS data from 2006 to 2011 | 13-15 | Tobacco | Gender- male, Being a student, Exposure to SHS (second hand smoke) | Moderate |
| 33 | Harrabi et al, 2009 | Predictors of smoking initiation among schoolchildren in Tunisia: a 4 year cohort study | Tunisia | 441 | It is a cohort study surveying, prospectively for 4 years, 453 students attending schools in Sousse city. Baseline data were obtained as a part of a randomized investigation about the cardiovascular diseases risk factors among 13-15-year-old pupils attending schools in Sousse city (Tunisia) in 1999. The total initial number of students was 789. After four years (2003), the questionnaire was filled in by pupils present in their classrooms at that day. | 13-15 | Tobacco | Gender-males, Socioeconomic status-pocket money value, Having friends who use substance, Tutors/teachers influence to smoke , Beliefs-smoking made one look cool, liberated ladies | Moderate |
| 34 | Maassen et al, 2004 | Smoking initiation among Gambian adolescents: social cognitive influences and the effect of cigarette sampling | The Gambia | 282 | The Gambia is divided into five divisions and two municipalities, including a total of 104 secondary schools (Jallow, 2001). We attempted to obtain representative data by selecting one school from every division/municipality, resulting in a total of seven schools. The sample included four governments, one private and two Roman Catholic schools (Latrikunda, Farafenni, Bansang, Kwinella, Banjul, Basse and Lamin), thereby representing the distribution of schools in The Gambia. Every school that was approached agreed to take part and all students agreed to take part in the study. One class per school was selected at random, with the average of 40 students per class. This resulted in a sample of 282 Gambian students, aged 14–18. | 14-18 | Tobacco | Depression aka. low mood, sad, hopeless, Lacking parental supervision, Having friends who use substance, Offered free, Parent works at the company making the substance, | Weak |
| 35 | Veeranki et al, 2015 | Prevalence and correlates of tobacco use among school-going adolescents in Madagascar | Madagascar | 1184 | The study data were obtained from the Madagascar Global Youth Tobacco Survey (GYTS) conducted in 2008 to obtain tobacco-related information from 1184 school-going adolescents aged 13–15 years and representing 296,111 youth. | 13-15 | Tobacco | Age –older adolescents, Gender-males, Exposure to product advertisement/ promotions, Liking product advertisement/ promotions, Having friends who use substance, Exposure to SHS | Strong |
| 36 | Panday et al 2003 | A qualitative study on the determinants of smoking behaviour among adolescents in South Africa | South Africa | 60 | The Group Areas Act of 1950 designated areas where people could reside such that people of the same race or ethnic group lived together. Schools were also divided by ethnicity such that ‘‘black’’ children attended school in their area, ‘‘whites’’ in their area and so forth. Despite the abolition of apartheid in 1994, most people still reside in previously defined group areas and schools, in the majority of cases, are ethnically homogenous. Six schools were purposively selected based on their locality, i.e. urban or rural, and their ethnicity, i.e. ‘‘black’’, ‘‘white’’ or ‘‘coloured’’. School selection was also based on the recommendations of the school nurses for this region and verified by the chief medical officer. One class of grade 8 students and one class of grade 9 students (13 – 14 years old) were randomly selected for each school. | 13-14 | Tobacco | Age-older adolescents, Gender-males, Having friends who use substance, Depression aka. low mood, sad, hopeless, Lacking parental supervision, Having friends who use substance, Ethnicity-racial discrimination against blacks and colored, Teachers/Tutors, Get away from other drugs, poverty-leads to unhealthy habits | Moderate |
| 37 | Rantao & Ayo-Yusuf 2012 | Dual use of cigarettes and smokeless tobacco among South African adolescents | South Africa | 1878 | The study sample frame consisted of all 31 school districts in the Limpopo Province (in the north of South Africa) and all the secondary schools in those school districts that were in the Department of Education’s database at the time of the study. Following a sample size determination of 20 schools, a 2- stage sampling design was used to produce a representative sample of grade 8 students from the schools in the province. The first stage of sampling involved a random selection of 22 of the 31 school districts to participate. | Mean age of 14 | Tobacco | Hazardous drug use, Hazardous alcohol use, depression aka. low mood, sad, hopeless, Anxiety a.k.a. worries, fears, using other drugs, living with someone who uses substances, | Strong |
| 38 | DeAtley et al 2020 | Risk and protective factors for whoonga use among adolescents in South Africa | South Africa | 200 | This paper utilizes data from Our Family Our Future, a pilot randomized controlled trial (RCT) designed to explore the acceptability and feasibility of an intervention to reduce adolescent HIV risk behaviors and depression. Data on whoonga use were derived from the baseline survey of N = 200 adolescents recruited for participation in the RCT. The RCT took place during 2015–2017 in a community in Cape Town, South Africa. An institutional review board approved all study protocols. Adolescents were recruited house-to-house within randomly selected enumeration areas. Adolescents were eligible to participate if they were between the ages of 13–15 years, lived in the household at least four days a week, confirmed that the adult was either a primary caregiver or parent, and met a threshold for elevated depressive symptoms. | 13-17 | Whoonga | Age (older adolescents), Hazardous drug use, Hazardous alcohol use | Weak |
| 39 | Reda et al 2012 | Prevalence and Determinants of Khat (Catha edulis) Chewing among High School Students in Eastern Ethiopia: A Cross-Sectional Study | Ethiopia | 1721 | The study was conducted in Harar own located 525 Km east of Addis Ababa, the capital city of Ethiopia. Harari Regional State is one of the nine regions in Ethiopia and Harar town is its capital city. The town has nine high schools from grades 9 to 12. The total number of students enrolled in these schools in the academic year at the time of this study was 6,523. | 15-19 | Khat | Age (older adolescents), Gender-males, Living with someone who uses substances, Having friends who use substances, Religion-prevents drug use, | Moderate |
| 40 | Oppong Asante 2019 | Cannabis and amphetamine use and its psychosocial correlates among school-going adolescents in Ghana | Ghana | 3632 | Data for this study were obtained from the Ghana Global School based Student Health Survey (GSHS) conducted in 2012 [21]. This survey was conducted through a partnership between the World Health Organization (WHO), the Center for Disease Control and Prevention (CDC), Middle Tennessee State University and the Ghana Education Service (GES). | 11-19 | Cannabis and Amphetamine | Suicide ideation, planning, and/or suicide attempt, Loneliness, Bullying, Truancy, Having family who uses substances, Smoked cigarettes, Parental knowledge of adolescent activities, Physically attacked, Number of close friends, | Moderate |
| 41 | Amoateng et al 2006 | Family predictors of adolescent substance use: the case of high school students in the Cape Metropolitan Area, Cape Town, South Africa | South Africa | 1800 | Data for this study came from a survey of approximately 1800 14-year-old (9th grade) and 17-year-old (11th grade) adolescents from 20 high schools in the Cape Town Metropolitan Area 1. | 14-17 | Alcohol and Tobacco | Gender- males, Socioeconomic status- rich parents more risk to use substance, Using other drugs, Parental knowledge of adolescent activities, Negative family variables (i.e. stress, hostility, and psychological control), Skin colour- blacks & coloured at more risk | Weak |
| 42 | Morojele et al 2016 | Tobacco and alcohol use among adolescents in South Africa: shared and unshared risks | South Africa | 736 | The participants were recruited via a household survey that was conducted in 2004. A three-stage sampling procedure was used to obtain the sample. This procedure involved sampling communities (census enumeration areas), followed by households, followed by adolescents in the households. One eligible adolescent (aged 12-17 years) was randomly selected from each household. | 12-17 | Alcohol and Tobacco | Age (older adolescents), Gender,-males Negative family variables (i.e. stress, hostility, and psychological control), Skin colour, Personal factors (i.e. self-deviance, violence, ego), School suspension | Strong |
| 43 | Getachew et al 2019 | Prevalence and risk factors for initiating tobacco and alcohol consumption in adolescents living in urban and rural Ethiopia | Ethiopia | 3697 | Ten rural and ten urban schools with respective rolls of 8304 and 13,528 were selected from a list of schools identified from the Addis Ababa City Administration, Oromia Regional State and Southern Nations, Nationalities, and People's Region (SNNPR) education offices. The rural schools were in Oromia state (eight) and SNNPR (two), which are located approximately 45e160 km from Addis Ababa, while the urban schools were in Addis Ababa. All schools that were invited to participate agreed and did so. There were 184 urban schools and 52 rural schools in this area, although we targeted schools that were accessible and representative of the area as whole. | 13-19 | Alcohol and Tobacco | Age (older adolescents), Gender-males, Having family who use substance, Religion- reduces the urge to use substances, Watching football- smoked more (EPL fan), Internet access at home-smoked more, | Weak |
| 44 | Morojele et al 2002 | Measurement of risk and protective factors for drug use and anti-social behavior among high school students in South Africa | South Africa | 123 | The participants comprised 92 (74 percent) males and 31 (25 percent) females (while one student’s gender was not indicated on the questionnaire). They were enrolled in Grade 8 (n = 81) and Grade 11 (n = 43) classes. Most of the students (79 percent) were “white,” while four were “Indian” (3 percent), six “black African” (5 percent), and 16 “colored” (13 percent) students. Their mean age was 14.33 years (S.D. = 1.48). The majority (95.2 percent; n = 118) reported that English was one of the languages that they spoke at home. | 13-16 | tobacco, alcohol, and cannabis use | Family management- poor family management results into high drug use, individual behavior- bad mannered at high risk and peer influence | Weak |
| 45 | Alwan et al 2011 | Association between substance use and psychosocial characteristics among adolescents of the Seychelles | Seychelles | 1432 | The data in this study come from the Global School based Health Survey (GSHS), which was conducted for the first time in the Seychelles in 2007 [31]. The GSHS, a school-based survey developed by the World Health Organization, the Centers for Disease Control and Prevention (Atlanta) and other international agencies, aims to provide a common methodology for collecting data on a broad set of risk behaviors and psychosocial characteristics among students worldwide (http://www.cdc.gov/gshs/questionnaire/index.htm). Schools were selected with probability proportional to school enrolment size. In Seychelles, all the 13 schools containing S1-S4 classes in the Seychelles were selected in the study. | 11-17 | tobacco, alcohol, and cannabis use | Age (older adolescents), Gender-males, Socioeconomic status- having pocket money, Suicide ideation, planning, and/or suicide attempt, Loneliness, Lacking parental supervision, Truancy, Number of close friends, Insomnia | Strong |
| 46 | Plüddemann et al 2008 | Adolescent methamphetamine use and sexual risk behaviour in secondary school students in Cape Town, South Africa | South Africa | 1561 | This study was part of a larger multi-site prospective panel study, the South Africa Tanzania (SATZ) project, which has been described elsewhere [13]. In each of three sites, Cape Town, Mankweng and Dar es Salaam, we conducted a cluster randomised controlled trial to investigate the effect of school-based HIV prevention programmes among grade 8 students. This grade was chosen to include students in the age range 12–14 years at baseline. | 12-17 | tobacco, alcohol and methamphetamine | Gender-males, being sexually active | Strong |
| 47 | Atilola et al 2013 | Beyond prevalence and pattern: problematic extent of alcohol and substance use among adolescents in Ibadan South-west Nigeria | Nigeria | 538 | The study was carried out in Ibadan, Nigeria. Ibadan is the third largest city in Nigeria with an estimated population of about 2.6 million spread over 11 local districts. Participants were sampled from 2 public schools within one of the 11 districts. The closest district to the domicile of the researchers was picked for convenience. All adolescents in the Senior Secondary Class 1 and 2 (corresponding with 10^th^ and 11th year of schooling) in the schools were sampled. | 15.1 ± 1.4 years | Multiple i.e. 4 or more substances | Age (older adolescents), Hazardous alcohol use, Gender-male, Lack of parental supervision, Having family who use substance, Poor academic performance, | Strong |
| 48 | Oshodi et al 2010 | Substance use among secondary school students in an urban setting in Nigeria: prevalence and associated factors | Nigeria | 420 | A total of ten out of the 45 secondary schools were selected for study using stratified sampling techniques. The schools were first stratified into two groups of public and private schools. Seven were randomly selected from among the public schools and three from the privately owned ones, to make a total of ten schools selected for study. From each of these schools selected for study, simple random sampling was used to select the number of subjects based on their proportions in relation to the whole sample size. | 11-20 | Multiple i.e. 4 or more substances | Hazardous drug use, Gender-males, Socioeconomic status- cheap substances that can be purchased, Depression aka. low mood, sad, hopeless, Using other drugs, Poor academic performance, Self-medication, Stimulants | Weak |
| 49 | Hamdulay & Mash 2011 | The prevalence of substance use and its associations amongst students attending high school in Mitchells Plain, Cape Town | South Africa | 438 | A sample size of at least 385 was calculated to give 5% precision with 95% confidence intervals. Twelve out of the 15 high schools agreed to participate, submitting a list of the number of Grade 8 and Grade 11 classes and the number of students per class. Classes were then randomly selected from each of the Grade 8 and Grade 11 lists until the required sample size was obtained. All the students in a selected class were invited to complete the questionnaire, a total of 545 students eventually being selected to allow for non-participation. All 12 high schools were represented in the selected grades, 226 students being selected from Grade 8 and 212 from Grade 11. Of the three high schools that refused to participate, one was undergoing a change of leadership and the other two did not respond to any forms of communication. Grade 8 and Grade 11 classes were selected to allow comparison with similar studies and because this covered the upper and lower ends of the adolescent age range. | Only mean age reported of 15.2 (SD 1.7). No age range reported | Multiple i.e. 4 or more substances | Age (older adolescents), Hazardous drug use, Hazardous alcohol use, Suicide ideation, planning, and/or suicide attempt, Using other drugs, Risky sexual behavior, Violence / physical abuse exposure, Pleasure/smell/taste, Being a student, | Moderate |
| 50 | Cumber & Tsoka-Gwegweni 2016 | Pattern and practice of psychoactive substance abuse and risky behaviours among street children in Cameroon | Cameroon | 399 | An analytical cross-sectional survey of street children aged 12 - 17 years was conducted from 1 January to 30 March 2015 in the three cities in Cameroon known to have large numbers of street children. The children were recruited using snowball sampling. Informed consent was obtained by the primary researcher using the language in which the children were most comfortable (English, French or a local language). Consent was obtained from all participants in all three cities. | 12-17 | Multiple i.e. 4 or more substances | Depression aka. low mood, sad, hopeless, Anxiety a.k.a. worries, fears, Lacking parental supervision, Having family who use substance, Having friends who use substance, Religion, Curiosity, | Weak |
| 51 | Embleton et al 2012 | Knowledge, Attitudes, and Substance Use Practices Among Street Children in Western Kenya | Kenya | 151 | Children and youth aged 10–19 classified as either children on the street or children of the street were recruited through street outreach to participate in a cross-sectional survey from May to July, 2011, at MTRH in Eldoret, Kenya. Street-involved children and youth were eligible to participate if they were between the ages of 10 and 19 and not currently enrolled in an educational institution and either (a) spending a portion or majority of their time on the street working or roaming while returning to sleep with family or a guardian at night (child on the street) or (b) having limited or no contact with family and spending both days and nights living and sleeping on the streets or in a shelter (child of the street). | 10-19 | Multiple i.e. 4 or more substances | Age (older adolescents), Hazardous drug use, Hazardous alcohol use, Gender-males, Socioeconomic status- money spent per day on drugs hence more money equals more drugs, Depression aka. low mood, sad, hopeless, Anxiety a.k.a. worries, fears, Using other drugs, Having family who use substance, Having friends who use substance, | Strong |
| 52 | Birungi et al 2021 | Substance use among HIV-infected adolescents in Uganda: rates and association with potential risks and outcome factors | Uganda | 479 | This study was undertaken at five youth HIV clinics in urban Kampala (the capital city of Uganda; the clinics were, Joint Clinical Research Centre and Nsambya Homecare Department) and in rural Masaka district (about 120 kilometres from Kampala; the clinics were, The AIDS Support Organization (TASO) clinic, Kitovu Mobile AIDS organization and The Uganda Cares clinic). A total sample of 1339 child/adolescent–caregiver dyads were recruited with each site contributing a varied number of participants. At the onset of the study, it was difficult to establish the actual number of active clients attending a given study clinics. We, therefore, allocated each study site an equal number of recruitment slots of 268 per study clinic. It was decided that during the course of the 6-month recruitment period, study participants would be enrolled continuously until a clinic’s allotted slots had been filled. It was also decided that if a study clinic experienced saturation (could no longer enrol new study participants), then the unused recruitment slots would be redistributed among the other clinics. | 12-17 | Multiple i.e. 4 or more substances | Age (older adolescents), Socioeconomic status- caregivers SES, Depression aka. low mood, sad, hopeless, Anxiety a.k.a. worries, fears, Using other drugs, | Weak |
| 53 | Ekop Eno et al 2019 | FACTORS ASSOCIATED WITH SUBSTANCE USE AMONG SECONDARY SCHOOL ADOLESCENTS IN GWAGWALADA AREA COUNCIL, ABUJA, NIGERIA | Nigeria | 1196 | Only adolescents who were aged 10 years to 18 years, and had given individual verbal assent as well as caregiver signed consent forms if less than 18 years old, and individually signed consent forms if 18 years old were included in the study. Any student absents from school or in the school bay during the time of the study was excluded | 10-18 | Multiple i.e. 4 or more substances | Gender- males, Marital status-parents single or married, Socioeconomic status-social class i.e. upper, middle lower, Using other drugs, Order of being born (first born, second born etc, | Strong |
| 54 | Kpozehouen et al 2015 | Factors associated with psychoactive substance use among Beninese adolescents | Benin | 451 | The study population consisted of adolescents aged 10 to 19 years, regardless of gender. All subjects aged 10 to 19 on the day of the survey and who had resided for at least six months in the villages or neighborhoods of the three communes of the Ouidah health zone were thus included in the study. These are the commune of Ouidah, Kpomassè and Tori-bossito (OKT). Not included were all subjects who had not given their consent to participate in the survey and all subjects who were unable to answer the questions themselves. | 10-19 | Multiple i.e. 4 or more substances | Age (older adolescents), Hazardous drug use, Hazardous alcohol use, Gender, Marital status, Socioeconomic status-, Depression aka. low mood, sad, hopeless, Anxiety a.k.a. worries, fears, Lacking parental supervision, Using other drugs, Having family who use substance, Living with someone who uses substances, Having friends who use substance, Adverse childhood events, Pleasure/smell/taste, | Weak |
| 55 | Birhanu et al 2014 | High prevalence of substance use and associated factors among high school adolescents in Woreta Town, Northwest Ethiopia: multi-domain factor analysis | Ethiopia | 684 | In Woreta town there are two government primary full cycle schools called Guaya and Dudumagn as well as one secondary and higher school where the study is conducted. The school teaches students from grade 9 to 12. As of the report of the town education sector, in the academic year of 2011/2012, there were a total of 3459 students from grades 9 to 12 attending their education. The study sample was recruited from grade 9 to 12 adolescent students (14 to 19 years age group) in Woreta secondary and high school. | 14-19 | Multiple i.e. 4 or more substances | Gender-males, Using other drugs , Having family who use substance, Having friends who use substance, Religion, Poor academic performance | Strong |
| 56 | Henry et al 2019 | Depressive symptoms, sexual activity, and substance use among adolescents in Kampala, Uganda | Uganda | 514 | After obtaining Institutional Review Board approval from Columbia University Medical Center and Makerere College of Health Sciences, data were abstracted from MMCAH’s baseline intake form. The 95-item intake form includes questions based on the “HEADDSS” Model for adolescent preventive health care (Home/health, Education/ employment, Activities, Drugs, Depression, Safety, Sexuality)7. Clinic staff or physicians completed the intake. Intake form during patients’ initial visit, and subsequently used it to guide patient interactions. Of the 702 patients presenting to the clinic between May 2013 and July 2015, some 558 intake forms were completed, 514 of which met the study age inclusion criteria. | 10-19 | Multiple i.e. 4 or more substances | Using other drugs, Having family who use substance, Having friends who use substance, Chronic illness, Loss of loved one, | Moderate |
| 57 | Peltzer, 2010 | Leisure time physical activity and sedentary behavior and substance use among in-school adolescents in eight African countries | Botswana, Kenya, Namibia, Se- negal, Swaziland, Uganda, Zambia, and Zimbabwe | 24593 | This study involved secondary analysis of existing data from the Global School-Based Health Survey (GSHS) from eight African countries (Botswana, Kenya, Namibia, Senegal, Swaziland, Uganda, Zambia, and Zimbabwe). All African countries from which GSHS datasets were publicly available were included in the analysis. Details and data of the GSHS can be accessed at <http://www.who.int/chp/gshs/methodology/en/index.html>. From all but one country, national samples were included, while from Zimbabwe, three areas were included: Harare, Bulawayo, and Manicaland. The aim of the GSHS is to collect data primarily from students of age 13 to 15 years. | 13-15 | Multiple i.e. 4 or more substances | Leisure/free time | Weak |
| 58 | Magidson et al 2017 | Psychosocial correlates of alcohol and other substance use among low-income adolescents in peri-urban Johannesburg, South Africa: A focus on gender differences | South Africa | 822 | Adolescents (age=16–18years; N= 822) were recruited from October 2008 to 2009 using a stratified sampling approach from four pre-identified, low socioeconomic, peri-urban areas outside of Johannesburg, South Africa: Soweto, Eldorado Park, Lenasia, and Brixton. Black adolescents were recruited from each of the 40 townships comprising Soweto, which were considered a stratum. Colored, Indian, and White adolescents were stratified by area from among Eldorado Park, Lenasia, and Brixton, respectively. Convenience sampling was employed within each stratum for all racial/ethnic groups. | 16-18 | Multiple i.e. 4 or more substances | Gender- females reported significantly greater depressive symptoms than males (p < .001). More males reported being sexually active than females (59% vs 39%; p < .0001) and low levels of parent–adolescent communication compared to females (59% vs 44%; p < .0001). Depression aka. low mood, sad, hopeless, Anxiety a.k.a. worries, fears, lacking parental supervision, having friends who use substance, Being sexually active, Violence / physical abuse exposure. | Moderate |
| 59 | Riva et al, 2018 | Prevalence and predictors of alcohol and drug use among secondary school students in Botswana: a cross-sectional study | Botswana | 1936 | A 72-item cross-sectional survey was administered to secondary school students at 17 secondary schools in Botswana’s capital city, Gaborone, and surrounding villages Lobatse, Molepolole, and Mochudi. We included all seven public senior secondary schools in our target area, and randomly selected ten of the 29 public junior secondary schools in blocks to include six schools in Gaborone, two in Molepolole and one each in of the smaller communities of Lobatse and Mochudi. At each junior secondary school, one study hall classroom per grade level (grades include Form 1 to 3, equivalent to grades 8 to 10 at American schools) was randomly selected to participate in the survey. At each senior secondary school, two Form 4 study hall classrooms and one Form 5 study hall classroom were randomly selected | 14-17 | Multiple i.e. 4 or more substances | availability of substance, Role model, Social, Vulnerability- | Strong |
| 60 | Ghuman & Hoque 2015 | Effect of religious beliefs on substance use among South African high school students | South Africa | 704 | We conducted a cross-sectional survey among adolescents aged 16-18 years from five high schools in the Emawaleni District of KwaZulu-Natal (KZN) during 2007. | 16-18 | Multiple i.e. 4 or more substances | Age (older adolescents), Religion, Ethnicity, | Weak |
| 61 | Taukoor et al 2017 | Substance use in adolescents with mental illness in Durban, South Africa | South Africa | 179 | 179 adolescents, aged 10 to 18 years old, with a mental disorder, and who were seen as new in- and out-patients at a psychiatric service in Durban from 1 July 2011 to 30 June 2013. Inclusion criteria All adolescents aged 10 to 18 years old presenting with first contact to mental health services were included in this study. Consecutive male and female adolescents meeting eligibility criteria were included in the study. The clinical diagnosis of a mental disorder as per DSM IV-TR (APA, 2000) had to be present for an adolescent to be included in the study. | 10-18 | Multiple i.e. 4 or more substances | Age (older adolescents), Gender- males, -, Depression aka. low mood, sad, hopeless, Anxiety a.k.a. worries, fears, Having family who use substance, Violence / physical abuse exposure, Ethnicity, Medical history, | Moderate |
| 62 | Doku et al 2012 | Socioeconomic differences in alcohol and drug use among Ghanaian adolescents | Ghana | 1195 | This study is based on a cross-sectional survey, which was conducted from June to August 2008 on health behaviours and lifestyles of school-aged adolescents in three administrative regions in Ghana. Thirty schools were randomly sampled, ten per region, from Eastern, Greater Accra and Volta Regions. The Ghana Education Service's School Health Programme register of schools in the country was the source of the sampling frame. | 12-18 | Multiple i.e. 4 or more substances | Hazardous alcohol use, Gender-males, socioeconomic status-rich to poor scales, Lacking parental supervision, Truancy, Poor academic performance, Family type-e.g. both parents dead, only one parent alive, both parents alive but not living together, nuclear family | Moderate |
| 63 | Plüddemann et al 2010 | Methamphetamine use, aggressive behavior and other mental health issues among high school students in Cape Town, South Africa | South Africa | 1561 | The school population was all high schools (N=54) in the South Educational District, one of four education management districts in the city of Cape Town. Fifteen schools were randomly selected from this population, such that the probability of selection was directly proportional to the number of students in the school. This district was believed to be the most affected by methamphetamine use, based on treatment demand data and newspaper and other anecdotal reports. Subsequently one class of approximately 30 students was randomly selected from each of grades 8 (majority aged 12–14), 9 (majority aged 14–15 and 10 (majority aged 15–17). Data were collected in July and August 2006. | 12-17 | Multiple i.e. 4 or more substances | Gender-males, Using other drugs, Being sexually active, | Strong |
